# Identification and determination of the absolute configuration of amorph-4-en-10β-ol, a cadinol-type sesquiterpene from the scent glands of the African reed frog *Hyperolius cinnamomeoventris*

**DOI:** 10.3762/bjoc.19.16

**Published:** 2023-02-16

**Authors:** Angelique Ladwig, Markus Kroll, Stefan Schulz

**Affiliations:** 1 Institute of Organic Chemistry, Technische Universität Braunschweig, Hagenring 30, 38106 Braunschweig, Germany

**Keywords:** Anura, chiral gas chromatography, enantioselective synthesis, GC/MS, semiochemicals

## Abstract

Hyperolid reed frogs are one of the few families of Anurans known to possess glands that emit volatile compounds used in chemical communication. *Hyperolius cinnamomeoventris*, a model species, possesses a gular gland on its vocal sac that emits chemicals, and sends visual and auditory signals during calling. Previous investigations have shown that the glandular compounds are typically macrocyclic lactones. However, in this work, we show that another major constituent of the male specific gland is (10*R*,1*S*,6*R*,7*R*,10*R*)-amorph-4-ene-10β-ol [(1*R*,4*R*,4a*R*,8a*S*)-4-isopropyl-1,6-dimethyl-1,2,3,4,4a,7,8,8a-octahydronaphthalen-1-ol]. This compound was synthesized for the first time and has the opposite configuration to amorph-4-ene-10β-ol known from plants. A short synthesis using an organocatalytic approach through a tandem Mannich/intramolecular Diels–Alder reaction led to a mixture of cadinols, which was used for the assignment of the natural cadinol structures and their stereoisomers.

## Introduction

**Figure 1:**
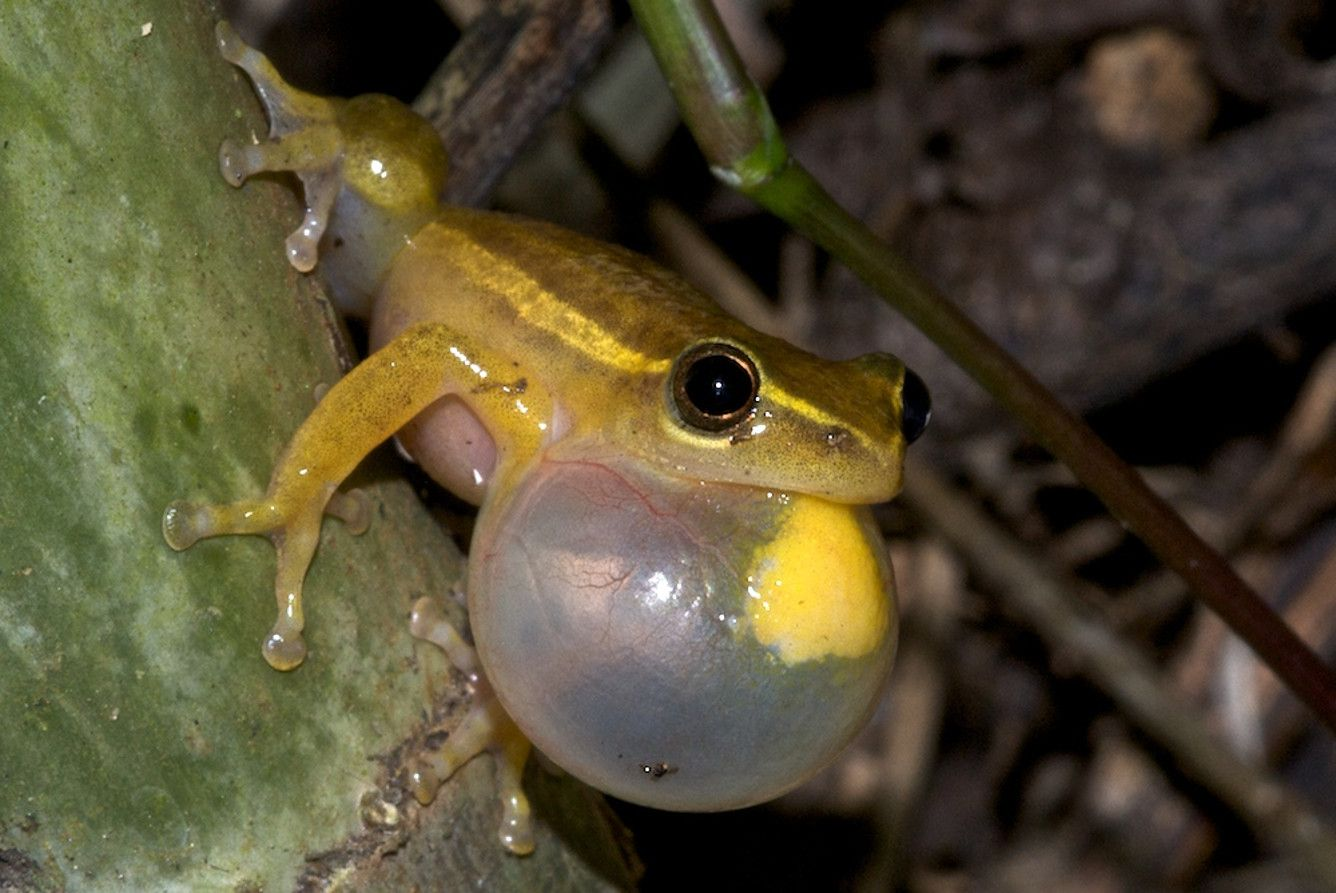
Calling male *Hyperolius cinnamomeoventris* with exposed vocal sac carrying the yellow gular gland. Figure 1 was reprinted from [2], I. Starnberger et al., “Take time to smell the frogs: vocal sac glands of reed frogs (Anura: Hyperoliidae) contain species-specific chemical cocktails”, Biological Journal of the Linnean Society, 2013, 110, 828–838, by permission of the Biological Journal of the Linnean Society published by John Wiley & Sons Ltd on behalf of The Linnean Society of London (© 2013 The Authors, Biological Journal of the Linnean Society; distributed under the terms of the Creative Commons Attribution-NonCommercial-NoDerivatives 4.0 International License, https://creativecommons.org/licenses/by-nc-nd/4.0/). This content is not subject to CC-BY-4.0.

*Hyperolius cinnamomeoventris* (Figure 1) is one of the largest species of reed frogs (Hyperoliidae), which are commonly found in Africa, south of the Sahara. Males of the Hyperoliidae possess a characteristic yellow gular patch on their vocal sac that also serves as a gland which emits volatile organic compounds during calling [1]. These courtship calls are trimodal, consisting of calls, yellow flashing signals, and volatile chemicals released from the gland [2]. The semiochemical signal, the glandular secretion, seems to be used for species recognition and mate choice. In the related frog family Mantellidae such functions of volatiles from males have been demonstrated [3], but no behavioral experiments involving semiochemicals have been performed so far within the hyperolid family.

**Figure 2:**
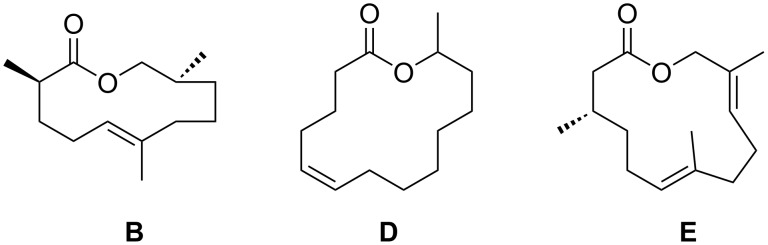
Macrolides identified in gular glands of male *Hyperolius cinnamomeoventris*.

**Figure 3:**
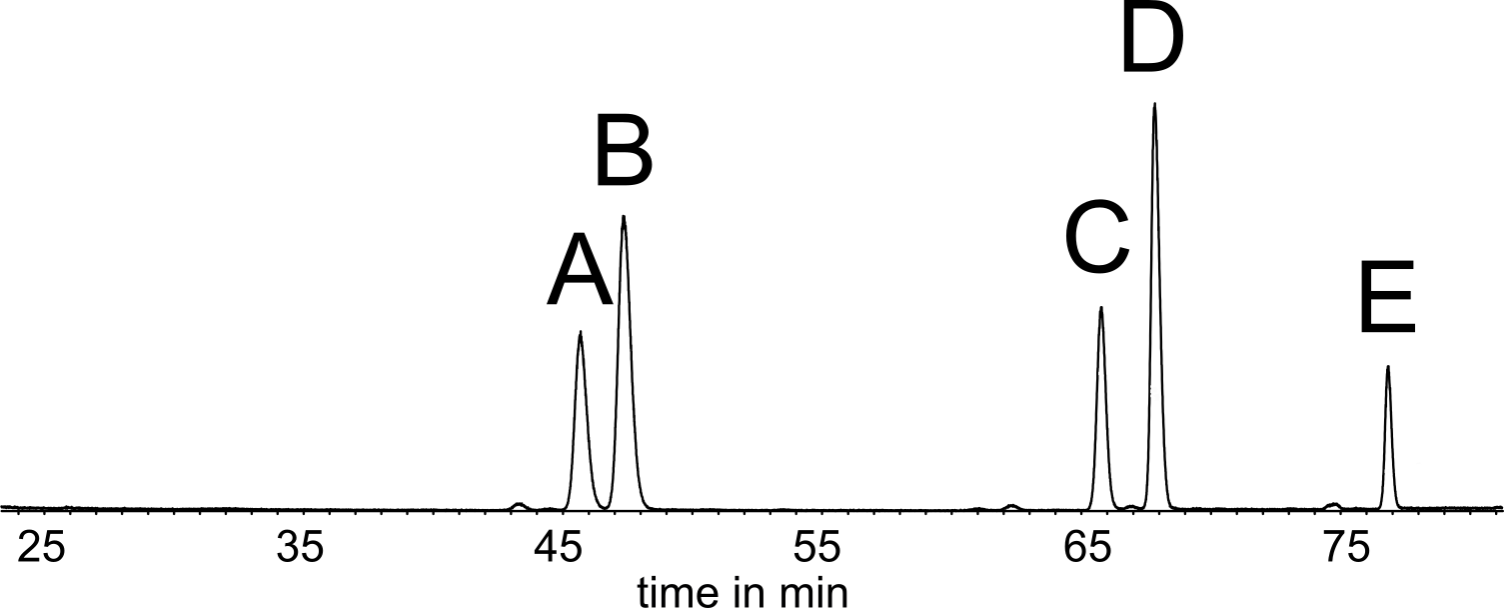
Total ion chromatogram (TIC) of a gular gland extract of *Hyperolius cinnamomeoventris* on a polar DB-wax GC phase. **A**, **C**: sesquiterpenes; **B**: cinnamomeoventrolide; **D**: (*Z*)-tetradec-5-en-13-olide; **E**: frogolide.

*H. cinnamomeoventris* also served as a model species for the investigation of the gular gland compound composition of hyperolids. The macrolides (*Z*)-tetradec-5-en-13-olide (**D**) [4], frogolide (**E**) [5], and cinnamomeoventrolide (**B**) [6] have been identified in earlier works as gular gland constituents of this species (Figure 2). Macrolides are commonly found as scent constituents of hyperolids, but also in scent-emitting femoral glands of the Mantellinae [7]. Contrary to mantellines, whose scent gland secretions are dominated by macrolides and secondary alcohol derivatives, hyperolid secretions additionally contain sesquiterpenes, such as constituents **A** and **C** (Figure 3).

As biological material is scarce and the amount of analytes is low, GC–MS trace analytical methods are performed to investigate extracts from the glands of individual frogs to identify their constituents. The analysis of MS and GC–IR data as well as gas chromatographic retention indices and microderivatization of extracts finally lead to structural proposals that have to be verified by synthesis. On this way a large variety of hyperolid and mantellid frog gland constituents have been identified [3–9]. Herein, we report on the structural elucidation of sesquiterpene **A**, including spectral analysis and synthesis to determine its constitution and absolute configuration.

## Results and Discussion

**Figure 4:**
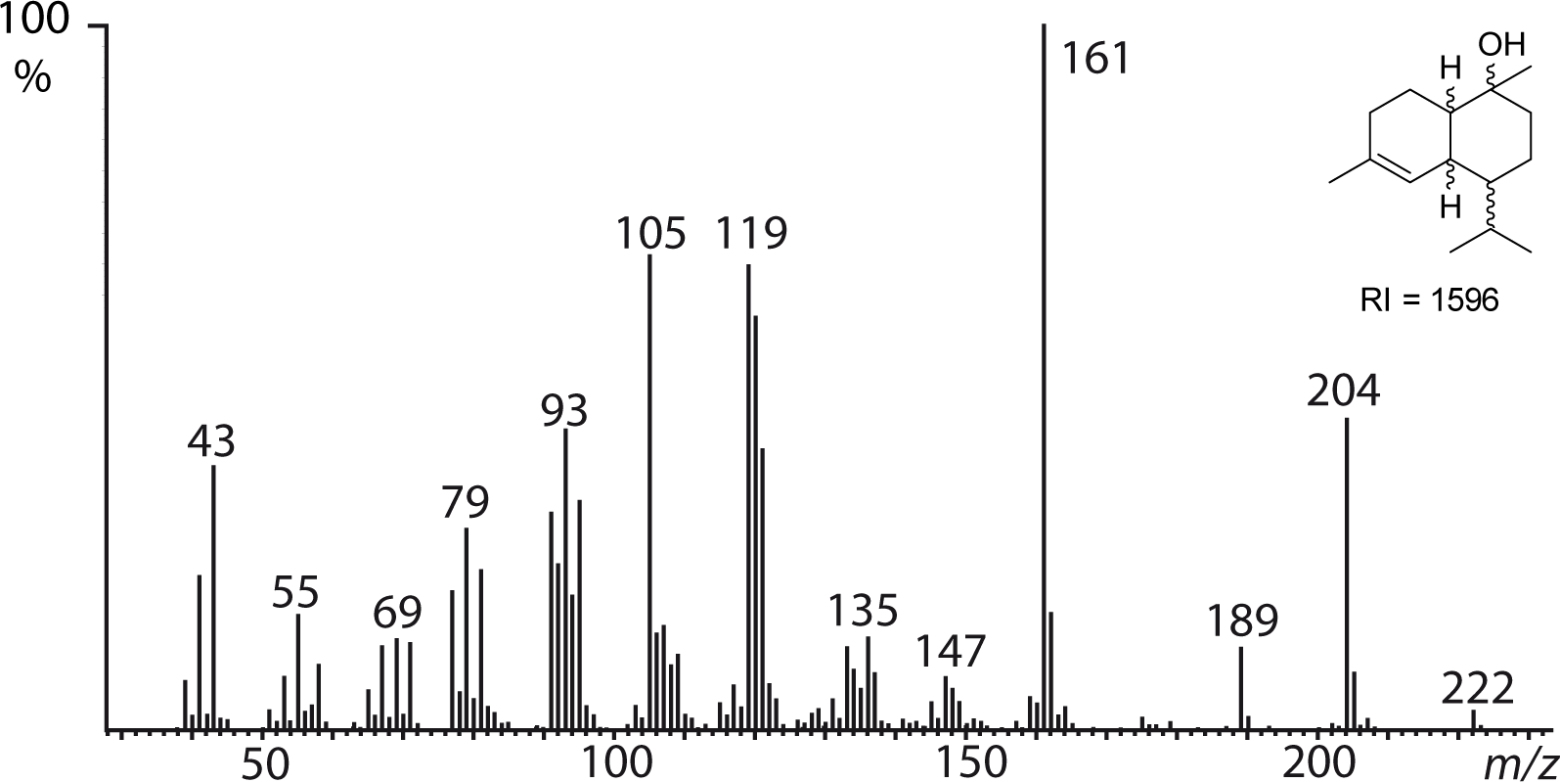
Mass spectrum of sesquiterpene **A** (*I* = 1596) from the gular gland extract of male *Hyperolius cinnamomeoventris,* identified to be amorph-4-en-10β-ol (**14**).

High-resolution mass spectrometry (HRMS) revealed both compounds **A** and **C** to be likely sesquiterpenes because of their molecular formula of C_15_H_26_O (*m/z* 222.1977, calcd for 222.1984) and C_15_H_22_O (*m/z* 216.1514, calcd for 216.1514), respectively, as well as the general appearance of their EI mass spectra. The mass spectrum of compound **A** (Figure 4) showed similarity to that of δ-cadinol (**12**) [10], but the linear gas chromatographic retention index [11] *I* = 1596 on an apolar DB-5 phase differed from the literature value of 1645 reported for compound **12** [12]. As no reference material was available, a problem hindering identification of sesquiterpenes in general, we planned to synthesize **12** and its seven diastereomers. Therefore, we adapted a synthesis of **12** originally developed by Taber and Gunn [13], using a Diels–Alder reaction as the key step, as it would allow access to several cadinol diastereomers, in line with a diversity-oriented synthetic plan.

**Scheme 1:**
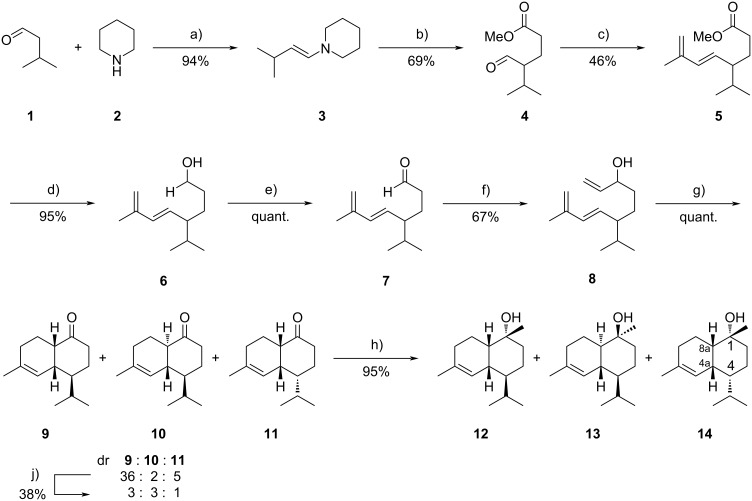
Racemic synthesis of cadinols modified from Taber and Gunn [13]. Conditions a) i) K_2_CO_3_ (0.35 equiv), 0 °C, 1 h, ii) rt, 2.5 h; b) i) methyl acrylate (1.3 equiv), MeCN, 90 °C, 42.5 h, ii) dest. H_2_O, AcOH, reflux, 1 h; c) i) diethyl (2-methylallyl)phosphonate (1.3 equiv), *n*-BuLi (1.3 equiv), THF, −78 °C, 35 min, ii) **4** (1.0 equiv), THF, −78 °C, 1 h, iii) rt, 1.5 h; d) i) LiAlH_4_ (1.2 equiv), Et_2_O, 0 °C, 15 min ii) rt, 45 min; e) IBX (3.0 equiv), EtOAc, reflux, 3 h; f) i) CH_2_=CHMgBr (1.5 equiv) Et_2_O, 0 °C, ii) rt, 20 min; g) IBX (3.0 equiv), EtOAc, reflux, 6 h; h) i) MeMgBr (1.5 equiv) Et_2_O, 0 °C, ii) rt, 30 min; j) NaOMe (25.0 equiv), MeOH, rt, 60 h.

The synthesis began with enamine formation of isovaleraldehyde (**1**) and piperidine (**2**) to give enamine **3** that was reacted in a Michael addition with methyl acrylate, affording aldehyde **4** (Scheme 1). Instead of the original Wittig reaction [13], a Horner–Wadsworth–Emmons reaction using diethyl (2-methylallyl)phosphonate and BuLi led to a higher yield and formation of the pure (*E*)-isomer **5**. The required phosphonate was cleanly obtained in 75% yield from triethyl phosphite and 3-chloro-2-methylpropene by addition of NaI [14]. Subsequent reduction of the ester with LiAlH_4_ and oxidation with IBX gave aldehyde **7** in 95% yield. Grignard addition of vinylmagnesium bromide afforded the alcohol **8**, which comprised the desired triene system for an intramolecular Diels–Alder reaction. Oxidation of **8** with IBX changed the electronic properties of the system implementing an electron-deficient double bond suitable for a heat-induced intramolecular Diels–Alder reaction. The higher reaction temperature compared to the original synthesis by Taber that used dichromate as an oxidant [13] led to a less diastereoselective reaction furnishing the three ketones **9**, **10**, and **11** in a ratio of 36:2:5. The *cis*-conformation of the decalin backbone of **9** and **11** originates from the *endo*-selectivity of the Diels–Alder reaction and the boat conformation of the transition state. A chair transition state is less preferred because of inherent non-bonding interactions of some hydrogens. This has been discussed in detail in the original publication by Taber and Gunn, favoring **9** as the main diastereomer [13]. Our results confirm these data. To increase the ratio of the minor diastereomers, the ketone mixture was treated with NaOMe, resulting in epimerization of **9**, arriving at a 3:3:1 mixture of **9**, **10**, and **11**. Unfortunately, a moderate amount of material was lost due to competing aldol reactions of the ketones. While compound **9** was epimerized to a large extent into **10**, the isomer **11** could not be converted into the respective trans-fused compound. This inseparable ketone mixture was then quantitatively converted into the target cadinols by addition of methylmagnesium bromide [15]. Major compounds were cedrelanol or τ-cadinol (**13**) and δ-cadinol (**12**) (for mass spectra see Supporting Information File 1). A minor diastereomer **14** was obtained in 9% yield after isolation by RP-HPLC using a LiChroPrep RP-18 phase because conventional column chromatography did not allow for good separation from the major products. Only one face of the carbonyl groups underwent nucleophilic attack, leading the formation of the three desired compounds.

The diastereomer **14** showed the same linear retention index *I* = 1596 and the same mass spectrum as **A**. After detailed NMR analysis, the relative configuration of the natural product could be determined. The most stable conformation was determined by calculation using force field methods (MMFF94 [16]) and is shown in Figure S2 of Supporting Information File 1. Key NOE couplings were observed between bridgehead H-8a, H-4a and H-4. The latter also couples with the methyl group at C-1, indicating a *cis*-decalin configuration with an equatorial isopropyl group and axial OH in compound **14**. A more detailed description showing the relevant NOESY correlations is given in Supporting Information File 1.

Compound **14** proved to be identical to amorph-4-en-10β-ol, which is a rare natural product, known from the wood oil of *Cryptomeria japonica* [17], the essential oil from *Aglaia odorata* [18], and from vetiver oil (*Vetiveria zizanioides*) [19]. Nevertheless, the absolute configuration of the natural compound remained unknown [18].

Having established the constitution and relative configuration of **A**, we then determined the absolute configuration. Controlling the stereogenic center C-4 of **4** would allow access to the respective enantiomers. Unfortunately, enantiomerically pure **4** is not easily available, in contrast to ketone **17**. This compound can be obtained in high optical purity using Jørgensen’s organocatalyst **16** [20–21]. In addition, such a synthetic approach would shorten the synthesis from eight to four steps and allow access to both enantiomers of the compounds **12**–**14**.

**Scheme 2:**
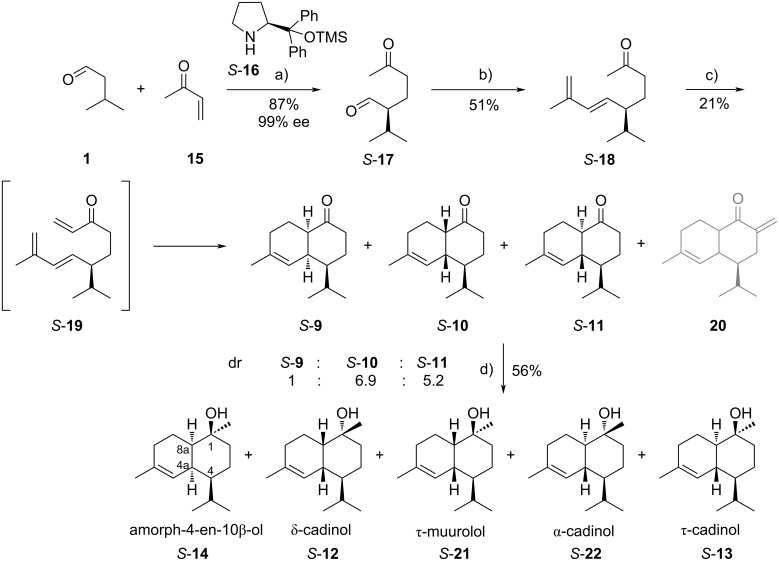
Enantioselective synthesis with (*S*)-Jørgensen’s organocatalyst *S*-**16**. Conditions: a) *S*-**16** (5 mol %), ethyl 3,4-dihydroxybenzoate (0.2 equiv), 4 °C, 36 h; b) i) diethyl (2-methylallyl)phosphonate (1.5 equiv), THF, −78 °C, 10 min, ii) *n*-BuLi (1.5 equiv), THF, −78 °C, 1 h, iii) *S-***17** (1.0 equiv), −78 °C, 10 min, rt, 8 h; c) i) formaldehyde (0.4 equiv), diisopropylamine (0.4 equiv), TFA (0.1 equiv), THF, reflux, 3 d, ii) maleic anhydride (1.2 equiv), reflux, 16 h; d) MeMgBr (3.0 equiv), THF, reflux, 4 h.

The synthesis started with an enantioselective Michael addition of aldehyde **1** to methyl vinyl ketone (**15**) catalyzed by (*S*)-Jørgensen’s organocatalyst *S*-**16**, to define the first stereogenic center at the isopropyl group, which becomes C-4 in the final products. Both *S*- and *R*-**16** were used to obtain the respective products in high ee and high yields (*R*-**17** = 87%, 98% ee, *S*-**17** = 87%, 99% ee) (Scheme 2) [20].

As in the racemic synthesis (Scheme 1), a HWE reaction using diethyl (2-methylallyl)phosphonate gave diene ketone *S*-**18** [13]. Here, we envisioned that a Mannich reaction would introduce the required α,β-unsaturated carbonyl system needed for the following intramolecular Diels–Alder reaction, that likely would proceed directly under these conditions. This concept proved to be difficult to achieve, but by optimization of different parameters of this Domino reaction, the required enantiomerically almost pure ketones **9**–**11** were obtained (Table 1). The Mannich reaction worked best using diisopropylammonium trifluoroacetate as the catalyst [22]. The isolation of *S*-**19** was attempted (Table 1, entry 1) but proved not to be necessary as the intramolecular Diels–Alder reaction proceeded readily during the reaction. Other conditions using an excess of formaldehyde, paraformaldehyde, or gaseous formaldehyde (Table 1, entries 2–4) were unsuccessful, mostly due to slow reaction, but a 37% solution of formaldehyde in water/methanol proved to be successful (Table 1, entries 5–10). Nevertheless, two problems were encountered. First, the Diels–Alder products **9**–**11** proved to be also active Mannich acceptors, leading to the unwanted unsaturated ketone **20**, a double Mannich product. To avoid this second addition, an excess or equimolar amounts of formaldehyde were avoided (Table 1, entries 6–10). The best result was achieved by adding only 0.4 equivalents formaldehyde to obtain products *S-***9**–**11** (Table 1, entries 9 and 10). The stereochemical descriptors *S* and *R* in compound numbers indicate the stereochemistry at C-4 in the cadinane system (Scheme 2) in the following discussion. Secondly, unreacted starting material **18** was not separable from the products by column chromatography. Therefore, maleic anhydride was added at the end of the reaction to form a Diels–Alder adduct with **18** that was readily separable from the target ketones. Following the optimization of reaction conditions, a change of solvent to THF was shown to be as equally effective as toluene (Table 1, entry 10).

**Table 1:** Screening of different reaction conditions with the (*S*)-Jørgensen’s organocatalyst *S*-**16** for the key Domino reaction, consecutive Mannich and intramolecular Diels–Alder reactions. DIA TFA: diisopropylammonium trifluoroacetate.

entry	reagent	base	solvent (reflux)	reaction time	result

1^a^	formaldehyde (11.8 equiv)	piperidine (0.13 equiv)	MeOH	1 d	*S*-**8** 23%
2^b^	paraformaldehyde (2.0 equiv, cracked in reaction mixture)	DIA TFA (2.0 equiv)	THF	10 d	*S*-**12**
3^b^	paraformaldehyde (1.0 equiv, cracked in reaction mixture)	DIA TFA (1.0 equiv)	THF	10 d	mixture
4^b^	paraformaldehyde (1.0 equiv cracked in a separate flask)	DIA TFA (1.0 equiv)	THF	10 d	mixture
5^b^	formaldehyde (37%) (1.0 equiv)	DIA TFA (2.0 equiv)	toluene	1 d	*S*-**12**
6^b^	formaldehyde (37%) (0.75 equiv)	DIA TFA (0.75 equiv)	toluene	12 h	*S*-**12**
7^b^	formaldehyde (37%) (0.6 equiv)	DIA TFA (0.6 equiv)	toluene	8 h	*S*-**12**
8^b^	formaldehyde (37%) (0.5 equiv)	DIA TFA (0.5 equiv)	toluene	8 h	*S*-**12**
9^b^	formaldehyde (37%) (0.4 equiv)	DIA TFA (0.4 equiv)	toluene	3 d	*S*-**9**–*S*-**11** 21%
10^b^	formaldehyde (37%) in methanol (0.4 equiv)	DIA TFA (0.4 equiv)	THF	3 d	*S*-**9**–*S*-**11** 21%

^a^With AcOH (0.22 equiv) [23], ^b^with TFA (0.1 equiv) [22].

The ketones *S*-**9**, *S*-**10**, and *S*-**11** were obtained in a ratio of 1:6.9:5.2, indicating a higher degree of epimerization at C-8a or a less selective Diels–Alder reaction compared to the racemic synthesis. Similar results were obtained within the *R*-series, starting with *R*-**17**. A final Grignard reaction using both ketone stereoisomeric mixtures with methylmagnesium bromide led to the *R*- and *S*-enantiomers of amorph-4-en-10β-ol (**14**), δ-cadinol (**12**), *epi*-α-muurolol or τ-muurolol (**21**), α-cadinol (**22**), and 10-*epi*-α-cadinol or τ-cadinol (**13**), respectively.

**Figure 5:**
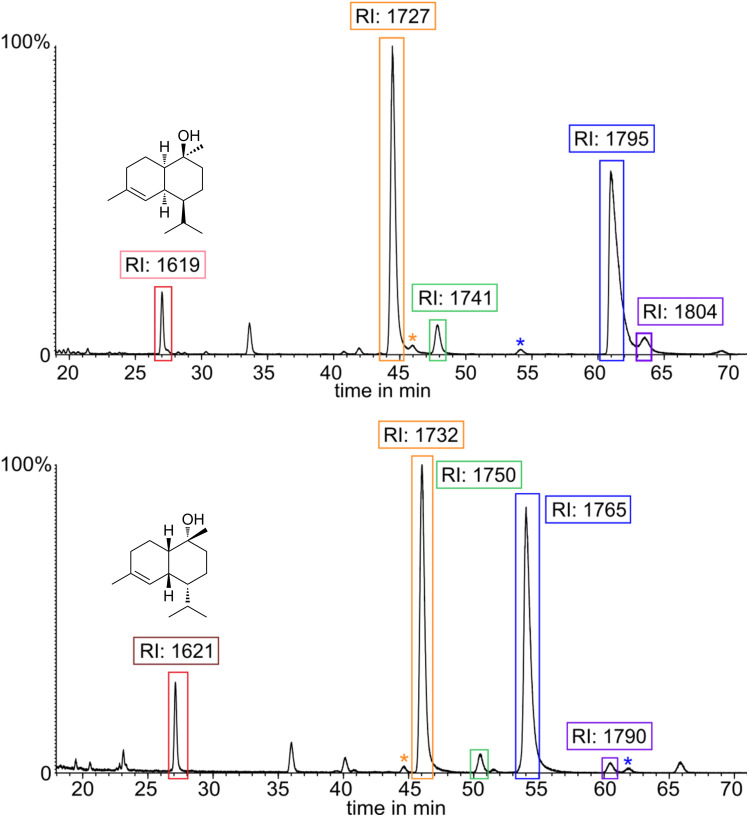
TIC and gas chromatographic Kovats retention indices RI [24] values determined on a Hydrodex β-6TBDM phase. The compounds are color-coded with the respective mass spectra in Figure 7 of each cadinol-type. Orange: δ-cadinol; blue: τ-cadinol; red: amorph-4-en-10β-ol; violet: α-cadinol. Upper trace: (4*S*)-diastereomers; lower trace: (4*R*)-diastereomers. *Corresponding enantiomers.

**Figure 6:**
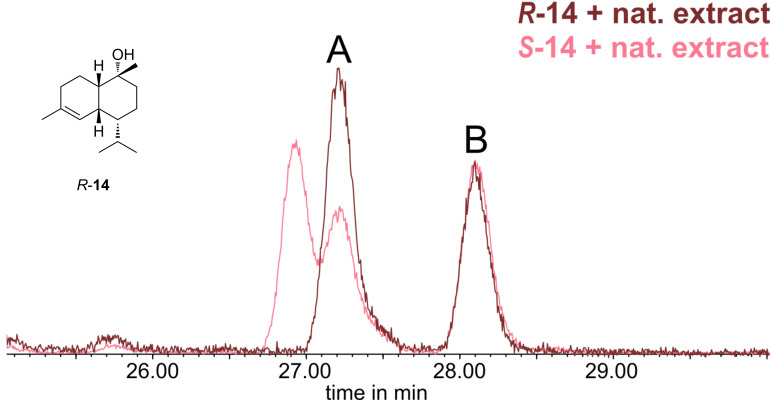
Coinjection of *R-***14** and *S-***14** with a gular gland extract of *Hyperolius cinnamomeoventris* performed with a Hydrodex β-6TBDM column. Compound **B** serves as relative standard to target compound **A**.

The isolation of product **14** proved unsuccessful due to the inseparability from the other cadinols. Nevertheless, with this material in hand, the absolute configuration of the sesquiterpene **A** was elucidated by enantioselective gas chromatography. The enantiomers of the alcohols could be separated on a Hydrodex β-6TBDM phase (Figure 5). This allowed to determine the absolute configuration of the sesquiterpene **A**. A coinjection of a gland extract with both the synthetic *R*- and *S*-samples would confirm the stereochemistry (Figure 6).

The coinjection of **A** with of *R-***14** showed only one peak with increased intensity, while two peaks were observed with *S-***14**. Therefore, the natural compound **A** is (1*R*,4*R*,4a*R*,8a*S*)-4-isopropyl-1,6-dimethyl-1,2,3,4,4a,7,8,8a-octahydronaphthalen-1-ol) (*R-***14**) or, according to the nomenclature used initially [17–18], (10*R*,1*S*,6*R*,7*R*,10*R*)-amorph-4-ene-10β-ol (see Figure S3 in Supporting Information File 1 for a comparison of compound numbering). To the best of our knowledge, this is the first determination of the absolute configuration of amorph-4-en-10β-ol (**14**) from a natural source. Alcohol **14** has been isolated before [17–18] or obtained by rearrangement from (+)-α-ylangene [25]. In the latter case the (4*S*)-stereoisomer of **14** was formed, as the isopropyl group is not affected by the rearrangement (see Figure S3 in the Supporting Information File 1). The same enantiomer was isolated from the wood oil of *Cryptomeria japonica* [17]. Therefore, the enantiomer *R-***14** from the frogs is different to the plant compound. This finding may point to a biosynthesis of **14** by the frogs themselves or by associated microorganisms, although uptake from the arthropod diet also may be possible, but less likely. The diet is varying. In addition, some arthropods can produce terpenes but they are not regarded as prolific producers of sesquiterpenes. The analysis of several *H. cinnamomeoventris* individuals from different locations showed a consistent composition of the gular gland blend, including always **14**. Diet-dependent uptake of compounds by frogs usually results in individual differences in compound composition, as has been observed, e.g., for skin alkaloids of Madagascan poison frogs [26]. Finally, experiments with mantellines showed that at least scent gland macrolides can be biosynthesized by the frogs [7], although the macrolides are produced from the fatty acid biosynthetic pathway.

**Figure 7:**
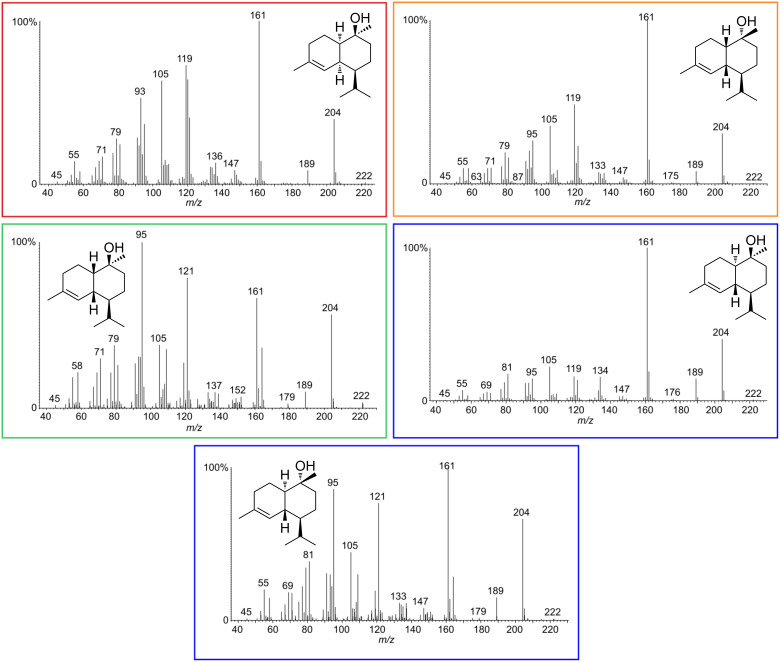
Mass spectra of each cadinol-type diastereomer. The box colors refer to the peaks and compounds in Figure 5.

The gas chromatographic separation obtained with the chiral phase also allowed the determination of the identity of the minor diastereomers formed during the reaction. This was not possible on a conventional DB-5-MS GC phase, because of overlapping peaks. Next to the major diastereomers **12**, **13** and **14**, *epi*-α-muurolol or τ-muurolol (**21**), and α-cadinol (**22**) were identified by their mass spectra [10,27] (Figure 7). After publication of this article, mass spectra and *I* of **12** and **14**, not present in the NIST 17 database will be made publically available in computer readable format through the open access mass spectra data base MACE [28].

## Conclusion

In this work, we have characterized compound **A** to be (10*R*,1*S*,6*R*,7*R*,10*R*)-amorph-4-ene-10β-ol or (1*R*,4*R*,4a*R*,8a*S*)-4-isopropyl-1,6-dimethyl-1,2,3,4,4a,7,8,8a-octahydronaphthalen-1-ol (*R-***14**) as part of the semiochemical mixture released by gular glands of the African reed frog *Hyperolius cinnamomeoventris*. This establishes the importance of sesquiterpenes for reed frogs, alongside macrocyclic lactones [2]. The total synthesis and characterization showed that this compound is the opposite enantiomer of **14** known from plants. This may indicate biosynthesis in the frog, but more work has to be performed to establish this. Furthermore, a short diversity-oriented synthesis based on the work of Taber and Gunn [13] enabled mass spectrometric and gas chromatographic data to be acquired, clarifying the identity and stereochemistry of several cadinols.

## Supporting Information

File 1 Numbering scheme, experimental procedures, ^1^H, ^13^C and 2D NMR spectra, and mass spectra.
