# Supplementary material for: Identification and determination of the absolute configuration of amorph-4-en-10β-ol, a cadinol-type sesquiterpene from the scent glands of the African reed frog Hyperolius cinnamomeoventris
Source: Beilstein J Org Chem. 2023 Feb 16;19:167–75. doi: 10.3762/bjoc.19.16 (PMC9940504; doi:10.3762/bjoc.19.16)
Supplement: File 1 — Numbering scheme, experimental procedures, 1H, 13C and 2D NMR spectra, and mass spectra. [file Beilstein_J_Org_Chem-19-167-s001.pdf]

BEILSTEIN JOURNAL OF ORGANIC CHEMISTRY

Supporting Information

for

Identification and determination of the absolute configuration of amorph-4-en-10β-ol, a cadinol-type sesquiterpene from the scent glands of the African reed frog *Hyperolius cinnamomeoventris*

Angelique Ladwig, Markus Kroll and Stefan Schulz

Beilstein J. Org. Chem. **2023,** *19,* 167–175. doi:10.3762/bjoc.19.16

Numbering scheme, experimental procedures, ^{1}H, ^{13}C and 2D NMR spectra, and mass spectra

License and Terms: This is a supporting information file under the terms of the Creative Commons Attribution License (https://creativecommons.org/licenses/by/4.0). Please note that the reuse, redistribution and reproduction in particular requires that the author(s) and source are credited and that individual graphics may be subject to special legal provisions.
The license is subject to the *Beilstein Journal of Organic Chemistry* terms and conditions: (https://www.beilstein-journals.org/bjoc/terms)

Table of contents

Mass spectra of compounds **12**, **13**, **14** S2
NOESY experiment analysis of compound **14**........ S3
General experimental protocol and characterization data S3
Detailed experimental protocols and characterization data S5
Racemic synthesis S5
Enantioselective synthesis S12
Special reaction setups S18
References........ S19
Spectral NMR data (^{1}H and ^{13}C NMR for compounds **12** and **14**) S20

Mass spectra of compounds 12, 13, and 14

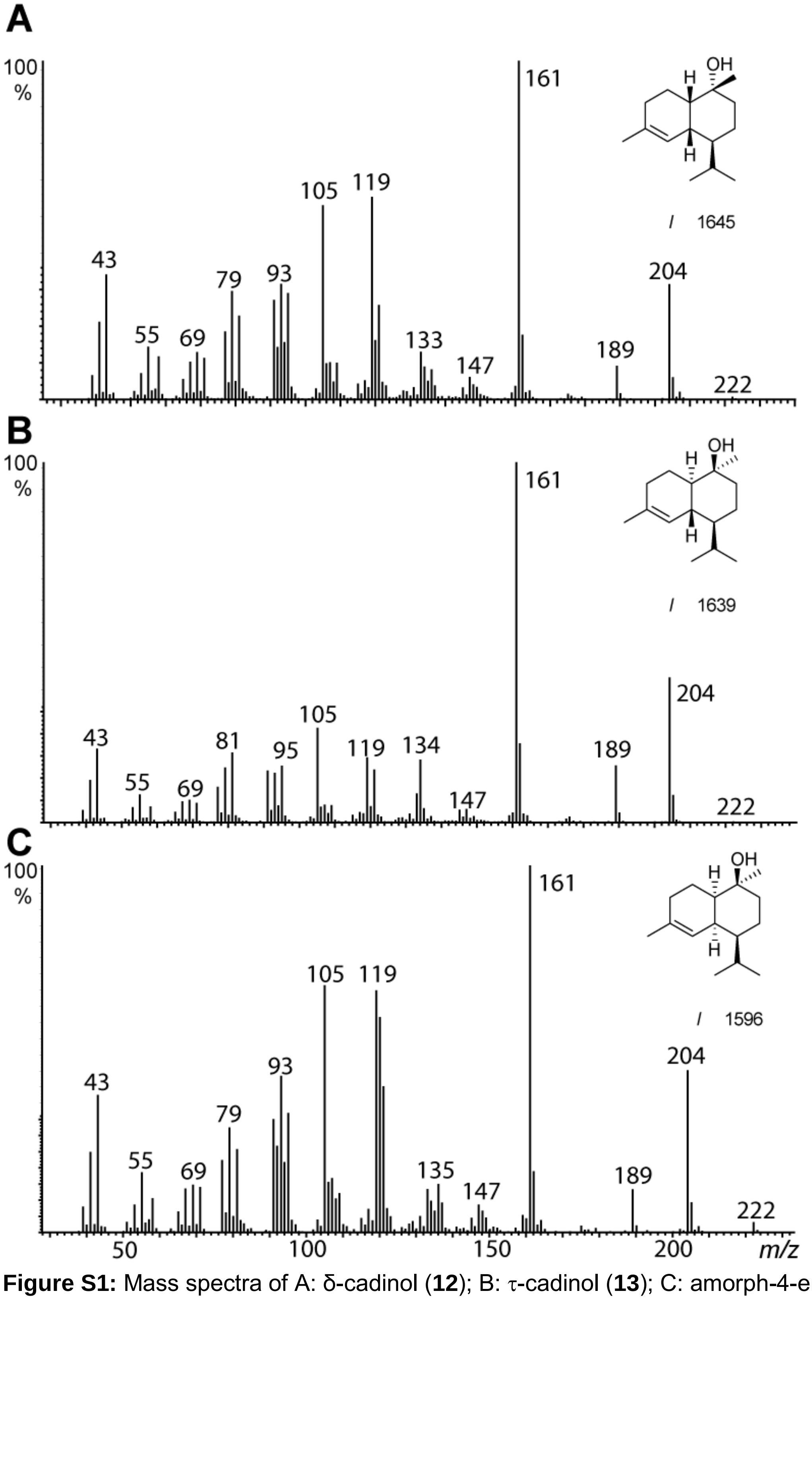

Figure S1: Mass spectra of A: δ-cadinol (**12**); B: τ-cadinol (**13**); C: amorph-4-ene-10β-ol (**14**).

NOESY experiment analysis of compound 14

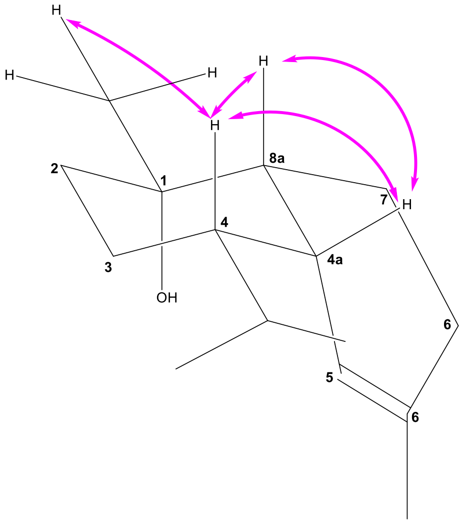

Figure S2: Important NOESY correlations of **14**. The magenta arrows mark the correlations used to determine the relative configuration. The most stable conformer of **14** was previously determined by calculation using the force field method MMFF94. The six-membered ring with C-1–C-4 has a chair conformation. The methylene substituent, which is larger than the vinyl group, is in the equatorial position, while the vinyl substituent is in axial position. The isopropyl group is also in equatorial position. The relative configuration could be determined from the observed cross signals in NOESY NMR experiments. Here, the protons H-4a and H-8a give a large correlation confirming the *cis*-decalin framework. The configuration of the substituents was obtained from the position of the protons H-4 and H-4a/H-8a. These gave two cross signals between H-8a/H-4 and H-4/H-4a, which means that the isopropyl group must be *anti* to the bridging protons. A cross signal from H-4 to the protons of the methyl group of C-1 provided the configuration at C-1. No cross signals could be observed between the OH group and H-8a/H-4a or H-4.

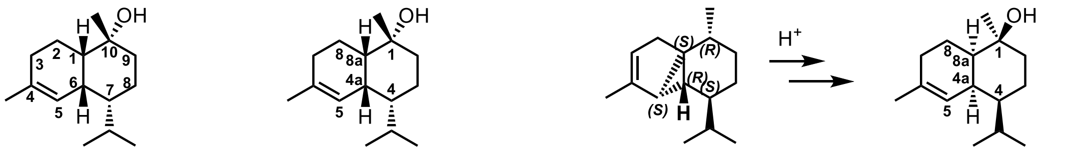

(1*S*,6*R*,7*R*,10*R*)-amorph-4-ene-10β–ol

(1*R*,4*R*,4a*R*,8a*S*)-4-isopropyl-1,6-dimethyl-1,2,3,4,4a,7,8,8a-octahydronaphthalen-1-ol

(+)-α-ylangene

(1*S*,4*S*,4a*S*,8a*R*)-4-isopropyl-1,6-dimethyl-1,2,3,4,4a,7,8,8a-octahydronaphthalen-1-ol

Figure S3: Nomenclature and numbering according to trivial terpene nomenclature (left) and IUPAC nomenclature (right). The rearrangement performed by Ohta et al. [S1] revealed the absolute configuration of **14** isolated by Nagahama et al. [S2].

General experimental protocol and characterization data

Chemicals were obtained from commercial suppliers and were used without further purification unless otherwise noted. All reactions were performed in oven-dried glassware under a nitrogen atmosphere, if not mentioned otherwise. Solvents were dried according to standard procedures. Column chromatography was performed on silica gel 60 (Fluka, particle size 0.040–0.063 mm, mesh 230–440 ASTM), reversed-phase column chromatography used LicroPrep RP-18, and thin-layer chromatography (TLC) was performed with Polygram® SIL G/UV 254 silica 60 plates (Macherey & Nagel, 0.20 mm thickness). Compounds were detected

with UV light (254 nm), anisaldehyde (5% anisaldehyde in ethanol with 1% sulfuric acid) or PMA (5% phosphomolybdic acid in ethanol) were used as staining solutions, followed by heating. NMR analyses were performed on Bruker Avance III HD 300 N (300 MHz for ^{1}H NMR, 75 MHz for ^{13}C NMR) and Avance III HD 500 (500 MHz for ^{1}H NMR, 125 MHz for ^{13}C NMR) spectrometers at room temperature. Chemical shifts are reported in ppm from tetramethylsilane as an internal standard ($\delta = 0$ ppm). Multiplicities of the protons are described as singlets (s), doublets (d), triplets (t), quartets (q), quintets (quint), sextets (sext), septets (sept), or multiplets (m). The multiplicities of the carbon atoms are described as primary (CH_3), secondary (CH_2), tertiary (CH), or quaternary (C_q). GC–MS analyses of synthetic products were performed with a combination of an Agilent Technologies 5977B gas chromatograph connected to an Agilent Technologies 8860 Series MSD. Mass spectrometry was performed in electron ionization mode (EI) with 70 eV. A HP-5 MS column (Agilent Technologies, 30 m length, 0.25 mm diameter, 0.25 µm film thickness, 350 °C) with helium as the carrier gas was used. The temperature program started at 50 °C, which was held for five min, followed by an increase with a rate of 20 °C/min to 320 °C. GC analyses on a chiral phase were performed using the combination of an Agilent Technologies 7890A with an Agilent Technologies 5975 Series MSD. A Hydrodex β-6TBDM column (Macherey & Nagel, 25 m length, 0.25 mm diameter, 0.25 µm film thickness, 230 °C) with helium as carrier gas used for the separation. The temperature program started at 50 °C and was held for five min. After that, the oven was heated to 120 °C at a rate of 25 °C/min and the temperature was maintained for 80 min. Finally, the temperature was increased to 230 °C at a rate of 10 °C/min for improvement of the peak shape and the final temperature was maintained for 5 min. The gas chromatographic retention indices were calculated using two different methods. While in case of the DB-5 phase linear retention indices *I* were calculated according to van den Dool and Kratz [S3] for temperature programmed conditions, in case of the Hydrodex phase the gas chromatographic retention index RI after Kovats [S4] was used, required for isothermal temperature programs. IR spectra were recorded on a Bruker Tensor 27 (diamond ATR). The intensities of the bands are given as strong (s), medium (m), weak (w) or broad (br). Optical rotations were determined with an MCP 150 polarimeter (Anton Paar) with a cell length of 10 cm and at 589 nm (*c* given in g/100 mL). A LAUDA Proline RP855 refrigerated thermostat was used for temperature control of reactions lasting several days. The bath was filled with a 0.9 mM Na_2CO_3 solution and the Michael additions were carried out at a constant temperature of 4 °C. Samples of gular glands of *Hyperolius cinnamomeoventris* were available [S5] due to permanent storage at −80 °C. The sample collection was performed between 2013 and 2014 under permits for species collection and export by the Rwanda Development Board.

Detailed experimental protocols and characterization data

Racemic synthesis

(*E*)-1-(3-Methylbut-1-enyl)piperidine (3)

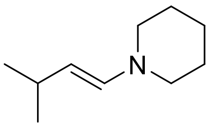

In a manner analogous to that of Storck et al. [S6], potassium carbonate (3.63 g, 26.3 mmol, 0.35 equiv) and piperidine (**2**, 15.95 g, 187.5 mmol, 2.50 equiv) were placed in a dried flask and cooled to 0 °C. Isovaleraldehyde (**1**, 6.46 g, 75.0 mmol, 1.00 equiv) was slowly added and the mixture stirred for 1 h under ice cooling. After stirring for a further 2.5 h at room temperature the resulting solid was filtered off and the product **3** (10.80 g, 70.5 mmol, 94%) was distilled at a head temperature of 65 °C at 4.0 mbar and obtained as a light yellow liquid.

^{1}H-NMR (400 MHz, $CDCl_3$, TMS): δ [ppm] = 5.79 (dd, 1 H, ^{3}J = 13.9 Hz, ^{4}J = 0.9 Hz), 4.37 (dd, 1 H, ^{3}J = 14.0 Hz, ^{3}J = 7.1 Hz), 2.74-2.71 (m, 4 H), 2.31-2.16 (m, 1H), 1.61-1.45 (m, 6 H), 0.97 (d, 6H, ^{3}J = 6.7 Hz).

^{13}C{^{1}H}-NMR (100 MHz, $CDCl_3$, TMS): δ [ppm] = 138.0 (1 C, CH), 109.6 (1 H, CH), 50.2 (2 C, CH_2), 29.5 (1 C, CH), 25.5 (2 C, CH_2), 24.4 (1 C, CH_2), 24.2 (2 C, CH_3).

IR (ATR, diamond): ν_{max} [cm^{-1}] = 2934 (s), 2858 (m), 2803 (m), 1651 (s), 1384 (m), 1233 (m), 1118 (s), 1038 (w), 1004 (m), 937 (s).

EI-MS (70 eV): *m*/*z* [%] = 153 (16, [M]$^{+}$), 138 (100), 122 (3), 110 (9), 94 (2), 82 (4), 69 (3), 55 (6), 41 (11).

Methyl 4-formyl-5-methylhexanoate (4)

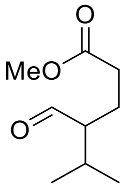

In a manner similar to that of Pettit et al. [S7], enamine **3** (10.63 g, 69.4 mmol, 1.0 equiv) and methyl acrylate (7.76 g, 90.2 mmol, 1.3 equiv) were reacted for 42.5 h in acetonitrile (105 mL) heated to reflux at 90 °C. Then, water (30 mL) and concentrated acetic acid (5 mL) were added and the mixture was heated to reflux for another hour. The reaction mixture was washed with NaCl solution (50 mL), the aqueous phase was separated and the organic phase dried over $MgSO_4$. The solvent was removed and the product was then distilled at 79 °C at 8.2 mbar. A colorless liquid (**4**, 8.27 g, 48.0 mmol, 69%) was obtained.

^{1}H-NMR (400 MHz, $CDCl_3$, TMS): δ [ppm] = 9.65-9.64 (m, 1 H), 3.67 (s, 3 H), 2.42-2.35 (m, 1 H), 2.29-2.21 (m, 2 H), 2.17- 1.90 (m, 1 H), 1.83-1.74 (m, 2 H), 1.01 (d, 3 H, ^{3}J = 6.9 Hz), 0.97 (d, 3 H, ^{3}J = 6.9 Hz).

^{13}C{^{1}H}-NMR (100 MHz, $CDCl_3$, TMS): δ [ppm] = 204.8 (1 C, CH), 173.5 (1 C, C_q), 57.4 (1 C, CH), 51.6 (1 C, CH), 31.9 (1 C, CH_2), 28.3 (1 C, CH_3), 20.7 (1 C, CH_2), 20.2 (1 C, CH_3), 19.4 (1 C, CH_3). ^{1}H- and ^{13}C-NMR data match those of Amat et al. [S8].

IR (ATR, Diamond): ν max [cm^{-1}] = 2960 (w), 2876 (w), 2717 (w), 1722 (s), 1437 (w), 1196 (w), 1170 (m).

EI-MS (70 eV): *m/z* [%] = 154 (2, $[M-H_2O]^+$), 144 (9, $[M-C_2H_4]^+$), 125 (13), 111 (5), 97 (12), 85 (13), 74 (100), 69 (30), 59 (12), 55 (28), 43 (32), 41 (30).

Diethyl (2-methallyl)phosphonate

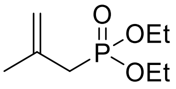

By an improved method of Nishikawa et al. [S9] triethyl phosphite (8.574 mL, 8.308 g, 50.0 mmol, 1.0 equiv), 3-chloro-2-methylpropene (5.355 mL, 4.980 g, 55.0 mmol, 1.1 equiv), and sodium iodide (1.124 g, 7.5 mmol, 0.15 equiv) were placed in a high pressure vessel for 42 h and heated to reflux at 90 °C. The reaction mixture was extracted three times with diethyl ether, washed with brine and water, dried over $MgSO_4$ and the solvent was removed under reduced pressure. The product (7.23 g, 37.6 mmol, 75%) was obtained after column chromatography as a light brown liquid.

DC [silica-gel, *n*-pentane/diethyl ether (1:6)]: R_f = 0.3 [PMA = blue].

^{1}H-NMR (400 MHz, $CDCl_3$, TMS): δ [ppm] = 4.98 – 4.86 (m, 2H, CH_2), 4.18 – 4.03 (m, 4H, CH_2), 2.59 (dd, ^3J = 22.4 Hz, ^3J = 1.0 Hz, 2H, CH_2), 1.89 (dd, ^3J = 3.0 Hz, ^3J = 1.2 Hz, 3H, CH_3), 1.32 (t, ^3J = 7.1 Hz, 6H, CH_3).

^{13}C{^{1}H}-NMR (100 MHz, $CDCl_3$, TMS): δ [ppm] = 136.2 (C_q), 115.3 (CH_2), 61.8 (CH_2), 36.1 (CH_2), 34.8 (CH_2), 23.7 (CH_3), 16.5 (CH_3), 16.4 (CH_3). ^{1}H- and ^{13}C-NMR data match those of Nishikawa *et al.* [S9].

^{31}P{^{1}H}-NMR (162 MHz, $CDCl_3$, H_3PO_4): δ [ppm] = 27.7 (s).

IR (solid phase): ν max [cm^{-1}] = 3082 (w), 2983 (m), 2948 (m), 2909 (m), 2869 (w), 1651 (m), 1418 (w), 1445 (m), 1393 (m), 1365 (w), 1288 (m), 1248 (s), 1216 (m), 1164 (m), 1100 (m), 1056 (s), 1029 (s), 965 (s), 892 (m), 840 (m), 773 (w).

EI-MS (70 eV): *m/z* [%] = 192 (39), 163 (20), 149 (18), 136 (98), 135 (26), 124 (24), 109 (28), 97 (100), 96 (99), 91 (21), 83 (37), 82 (72), 81 (66), 65 (29), 56 (20), 55 (65), 53 (22), 39 (30).

Methyl (*E*)-4-isopropyl-7-methylocta-5,7-dienoate (5)

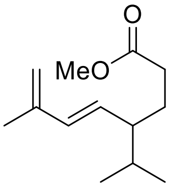

In a manner analogous to Nishikawa et al. [S9], diethyl (2-methylallyl)phosphonate (11.90 g, 61.9 mmol, 1.3 equiv) in THF (200 mL) at −78 °C was mixed with *n*-BuLi in hexanes (1.6 M, 39 mL, 61.9 mmol, 1.3 equiv) and the mixture stirred for 35 min at −78 °C. The aldehyde **4** (8.18 g, 47.6 mmol, 1 equiv) in THF (100 mL) was then added over 10 min. The reaction mixture was stirred for one hour at −78 °C and then for 1.5 h at room temperature. The reaction was quenched by the addition of sat. NH_4Cl solution (50 mL) and water (50 mL). The organic layer was separated and the aqueous phase was extracted three times with diethyl ether

(50 mL each). After drying over $MgSO_4$ and removal of the solvent, the product was separated by column chromatography [silica-gel, *n*-pentane/diethyl ether 15:1] to give **5** as a colorless liquid (4.59 g, 21.9 mmol, 46%).

DC [silica-gel, *n*-pentane/diethyl ether (15:1)]: R_f = 0.25 [PMA = purple].

^{1}H-NMR (300 MHz, $CDCl_3$, TMS): δ [ppm] = 6.07 (d, 1 H, ^3J = 15.6 Hz), 5.4 (dd, 1 H, ^3J = 15.6 Hz, ^3J = 9.3 Hz), 4.88 (s, 2 H), 3.65 (s, 3 H), 2.36-2.14 (m, 2 H), 1.83 (s, 3 H), 1.83-1.82 (m, 2 H), 1.61-1.52 (m, 2 H), 0.89 (d, 3 H, ^3J = 6.8 Hz), 0.85 (d, 3 H, ^3J = 6.8 Hz).

^{13}C{^{1}H}-NMR (75.5 MHz, $CDCl_3$, TMS): δ [ppm] = 174.5 (1 C, C_q), 141.9 (1 C, C_q), 134.6 (1 C, CH), 131.7 (1 C, CH), 114.7 (1 C, CH_2), 51.4 (1 C, CH), 49.4 (1 C, CH), 32.6 (1 C, CH_2), 32.3 (1 C, CH_3), 27.5 (1 C, CH_2), 20.7 (1 C, CH_3), 19.2 (1 C, CH_3), 18.7 (1 C, CH_3). ^{1}H- and ^{13}C-NMR data match those of Nishikawa *et al.* [S9].

IR (ATR, Diamond): ν $_{max}$ [cm^{-1}] = 3082 (w), 2956 (m), 2873 (w), 1739 (s), 1609 (w),1436 (w), 1162 (m), 1016 (m), 833 (m).

EI-MS (70 eV): *m/z* [%] = 210 (41 [M]$^+$), 195 (6), 179 (7), 163 (9), 154 (5), 142 (10), 135 (40), 132 (52), 107 (58), 93 (100), 79 (39), 67 (12), 55 (14), 51 (4), 41 (22).

(*E*)-4-Isopropyl-7-methylocta-5,7-dien-1-ol (6)

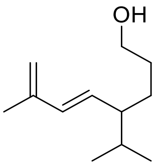

$LiAlH_4$ (0.58 g, 15.3 mmol, 1.20 equiv) was placed in a dried flask under a nitrogen atmosphere and diethyl ether (50 mL) was added. Then, ester **5** (2.68 g, 12.8 mmol, 1.00 equiv) dissolved in diethyl ether (20 mL) was added under ice cooling. The mixture was stirred for 15 min under ice cooling and then stirred for 45 min at room temperature. The reaction was quenched by the addition of sat. NH_4Cl solution and water. The phases were separated and the aqueous phase was extracted three times with diethyl ether (30 mL each). The combined organic phases were dried over $MgSO_4$ and then filtered. The solvent was removed, leading to product **6** (2.21 g, 12.1 mmol, 95%), obtained as a colorless liquid. The material was pure enough to be used without further purification in the next step [S10].

^{1}H-NMR (400 MHz, $CDCl_3$, TMS): δ [ppm] = 6.08 (d, 1 H, ^3J = 15.6 Hz), 5.40 (dd, 1 H, ^3J = 15.7 Hz, ^3J = 9.4 Hz), 4.90-4.85 (m, 2 H), 3.64-3.60 (m, 2 H), 1.87-1.81 (m, 1 H), 1.83 (dd, 3 H, ^4J = 1.3 Hz, ^4J = 1.0 Hz), 1.75-1.71 (m, 2 H), 1.68-1.14 (m, 4 H), 0.88 (d, 3 H, ^3J = 6.8 Hz), 0.84 (d, 3 H, ^3J = 6.8 Hz).

^{13}C{^{1}H}-NMR (100 MHz, $CDCl_3$, TMS): δ [ppm] = 144.3 (1 C, C_q), 134.1 (1 C, CH), 132.9 (1 C, CH), 114.4 (1 C, CH_2), 63.4 (1 C, CH_2), 49.6 (1 C, CH), 32.4 (1 C, CH), 31.2 (1 C, CH_2), 28.7 (1 C, CH_2), 20.9 (1 C, CH_3), 19.2 (1 C, CH_3), 18.9 (1 C, CH_3).

IR (ATR, Diamond): ν $_{max}$ [cm^{-1}] = 3318 (br. s), 2955 (m), 2870 (m), 1608 (w), 1454 (m), 1369 (m), 1055 (m), 968 (s), 882 (s), 543 (w).

EI-MS (70 eV): *m/z* [%] = 182 (14) [M]$^+$, 167 (2) [M-CH_3]$^+$, 149 (3), 139 (8), 121 (26), 113 (37), 105 (16), 93 (100), 79 (56), 67 (21), 55 (24), 41 (29).

(*E*)-4-Isopropyl-7-methylocta-5,7-dienal (7)

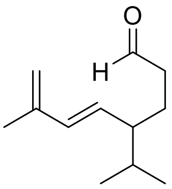

The alcohol **6** (2.21 g, 12.13 mmol, 1.0 equiv) was mixed with IBX (10.19 g, 36.39 mmol, 3.0 equiv) in ethyl acetate (80 mL) and heated to reflux for 3 h. Subsequently, unreacted IBX was removed via a short filter column (EtOAc as eluent). The aldehyde **7** (2.20 g, 12.13 mmol, quant.) was quantitatively obtained.

^{1}H-NMR (300 MHz, $CDCl_3$, TMS): δ [ppm] = 9.75 (t, 1 H, ^3J = 1.5 Hz), 6.07 (d, 1 H, ^3J = 15.7 Hz), 5.34 (dd, 1 H, ^3J = 15.6 Hz, ^3J = 9.4 Hz), 4.90-4.85 (m, 2 H), 1.90-1.76 (m, 3 H), 1.83 (dd, 3 H, ^4J = 1.3 Hz, ^4J = 0.8 Hz), 1.69-1.47 (m, 3 H), 0.90 (d, 3 H, ^3J = 6.6 Hz), 0.85 (d, 3 H, ^3J = 6.6 Hz).

^{13}C{^{1}H}-NMR (75.5 MHz, $CDCl_3$, TMS): δ [ppm] = 203.0 (1 C, CH), 142.0 (1 C, C_q), 135.0 (1 C, CH), 131.8 (1 C, CH), 115.0 (1 C, CH_2), 49.4 (1 C, CH), 42.6 (1 C, CH_2), 32.5 (1 C, CH), 24.8 (1 C, CH_2), 20.8 (1 C, CH_3), 19.3 (1 C, CH_3), 18.9 (1 C, CH_3).

IR (ATR, Diamond): ν_{max} [cm^{-1}] = 3081 (m), 2958 (s), 2873 (s), 2825 (s), 2725 (s), 1724 (s), 1646 (w), 1610 (s), 1410 (s), 1386 (s), 1367 (s), 1248 (m), 1050 (m), 972 (s), 886 (s).

EI-MS (70 eV): *m*/*z* [%] = 180 (3) [M]$^+$, 165 (2), 162 (2), 151 (1), 147 (3), 137 (23) ,136 (43), 121 (33), 109 (30), 93 (100), 79 (34), 67 (26), 55 (16), 41 (28).

(*E*)-6-Isopropyl-9-methyldeca-1,7,9-trien-3-ol (8)

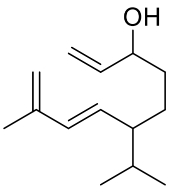

Aldehyde **7** (2.20 g, 12.13 mmol, 1.0 equiv) was dissolved in dry diethyl ether (50 mL) and vinylmagnesium bromide in THF (1.0 M, 18.2 mL, 18.2 mmol, 1.5 equiv) was added under ice cooling and the solution was stirred for 20 min at room temperature. The reaction was terminated by the addition of cold sat. NH_4Cl solution (30 mL). The phases were separated and the aqueous phase was extracted three times with diethyl ether (30 mL each). The combined organic phases were dried over $MgSO_4$, then filtered and the solvent was removed. After column chromatographic purification [silica-gel, *n*-pentane/diethyl ether 5:1] the alcohol **8** (1.68 g, 8.10 mmol, 67%) was obtained as a clear liquid [S11].

DC [silica-gel, *n*-pentane/diethyl ether (5:1)]: R_f = 0.26 [PMA = purple].

^{1}H-NMR (600 MHz, $CDCl_3$, TMS): δ [ppm] = 6.07 (d, 1 H, ^3J = 15.4 Hz), 5.88-5.82 (m, 1H), 5.40 (dd, 1 H, ^3J = 15.6 Hz, ^3J = 9.2 Hz), 5.23-5.19 (m, 1 H), 5.11-5.08 (m, 1 H), 4.90-4.85 (m, 2 H), 4.07 (dt, 1 H, ^3J = 6.5 Hz, ^3J = 6.5 Hz) 1.86-1.76 (m, 1 H), 1.83 (dd, 3 H, ^4J = 1.9 Hz, ^4J = 1.0 Hz), 1.65-1.33 (m, 5 H), 0.88 (d, 3 H, ^3J = 6.8 Hz), 0.83 (d, 3 H, ^3J = 6.8 Hz).

$^{13}C\{^1H\}$-NMR (151 MHz, $CDCl_3$, TMS): δ [ppm] = 171.3 (1 C, C_q), 141.3 (1 C, CH), 134.2 (1 C, CH), 132.8 (1 C, CH), 114.9 (1 C, CH_2), 114.4 (1 C, CH_2), 73.5 (1 C, CH), 49.8 (1 C, CH), 35.4 (1 C, CH_2), 32.4 (1 C, CH), 28.3 (1 C, CH_2), 20.9 (1 C, CH_3), 19.2 (1 C, CH_3), 19.0 (1 C, CH_3).

IR (ATR, Diamond): ν_{max} [cm^{-1}] = 3341 (br. s), 2956 (m), 2870 (m), 1608 (w), 1455 (w), 1384 (w), 1369 (w), 990 (m), 968 (s), 919 (s), 882 (s), 686 (w).

EI-MS (70 eV): *m/z* [%] = 208 (2) $[M]^+$, 193 (6) $[M\text{-}CH3]^+$, 190 (4), 175 (9), 165 (18), 147 (34), 136 (53), 121 (70), 105 (66), 93 (100), 79 (83), 67 (49), 55 (51), 41 (54).

4-Isopropyl-6-methyl-3,4,4a,7,8,8a-hexahydronaphthalen-1(2*H*)-ones 9, 10, and 11

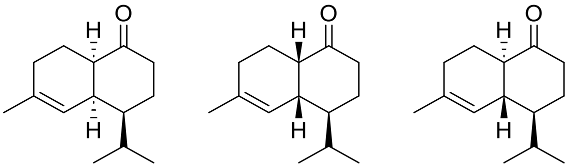

Alcohol **8** (1.68 g, 8.10 mmol, 1.0 equiv) was mixed with IBX (6.80 g, 24.30 mmol, 3.0 equiv) in ethyl acetate (50 mL) and stirred for 6 h under reflux. Subsequently, the mixture was filtered through a short pad of silica (ethyl acetate). The solvent was removed from the filtrate on a rotary evaporator. The three products **9**, **10** and **11** were quantitatively obtained as a colorless liquid, in a ratio of 36:2:5, and were used in the next step without further purification. The NMR data could be assigned to the individual compounds based on the ratio from the GC data. The ketones could not be separated by column chromatography [S12].

(4S,4aS*,8aR*)-4-Isopropyl-6-methyl-3,4,4a,7,8,8a-hexahydronaphthalen-1(2H)-one (**9**)*

^1H-NMR (600 MHz, $CDCl_3$, TMS): δ [ppm] = 5.25-5.21 (m, 1 H), 2.45-2.41 (m, 1 H), 2.39-2.35 (m, 1 H), 2.32-2.22 (m, 2 H), 2.01-1.84 (m, 2 H), 1.76-1.68 (m, 2 H), 1.62 (s, 3 H), 1.60-1.49 (m, 3 H), 1.46- 1.39 (m, 1 H), 1.00-0.96 (m, 3 H), 0.90-0.87 (m, 3 H).

$^{13}C\{^1H\}$-NMR (151 MHz, $CDCl_3$, TMS): δ [ppm] = 212.6 (1 C, C_q), 137.9 (1 C, C_q), 119.3 (1 C, CH), 47.5 (1 C, CH), 47.4 (1 C, CH), 41.6 (1 C, CH), 39.0 (1 C, CH_2), 28.6 (1 C, CH_2), 27.1 (1 C, CH), 26.3 (1 C, CH_3), 24.1 (1 C, CH_2), 23.0 (1 C, CH_2), 22.0 (1 C, CH_3), 21.1 (1 C, CH_3).

IR (solid phase): ν_{max} [cm^{-1}] = 2960 (s), 2920 (s), 2874 (s), 1706 (s), 1668 (m), 1474 (s), 1445 (s), 1385 (s), 1361 (s), 1325 (s), 1286 (s), 1269 (s), 1177 (s), 1160 (s), 1148 (s), 1141 (s), 1101 (s), 1067 (m), 1001 (s), 954 (s), 928 (m), 855 (s), 828 (m), 783 (m).

EI-MS (70 eV): *m/z* [%] = 206 (100) $[M]^+$, 191 (33) $[M\text{-}CH_3]^+$, 178 (10), 164 (39), 163 (38), 145 (65), 136 (58), 121 (77), 105 (25), 94 (64), 79 (70), 55 (40), 41 (39).

(4S,4aR*,8aS*)-4-Isopropyl-6-methyl-3,4,4a,7,8,8a-hexahydronaphthalen-1(2*H*)-one (**10**)*

^1H-NMR (600 MHz, $CDCl_3$, TMS): δ [ppm] = 5.31-5.29 (m, 1 H), 2.53-2.48 (m, 1 H), 2.45-2.41 (m, 1 H), 2.32-2.22 (m, 2 H), 2.10-2.01 (m, 1 H), 2.01-1.84 (m, 2 H), 1.83-1.76 (m, 2 H), 1.62 (s, 3 H), 1.60- 1.49 (m, 2 H), 1.46-1.39 (m, 1 H), 1.00-0.96 (m, 3 H), 0.90-0.87 (m, 3 H).

$^{13}C\{^1H\}$-NMR (151 MHz, $CDCl_3$, TMS): δ [ppm] = 214.7 (1 C, C_q), 135.1 (1 C, C_q), 124.3 (1 C, CH), 46.8 (1 C, CH), 45.2 (1 C, CH), 39.0 (1 C, CH), 38.3 (1 C, CH_2), 28.6 (1 C, CH_2), 26.9 (1 C, CH), 23.8 (1 C, CH_3), 23.7 (1 C, CH_2), 23.1 (1 C, CH_2), 21.6 (1 C, CH_3), 17.9 (1 C, CH_3).

IR (solid phase): ν max [cm^{-1}] = 3008 (m), 2959 (s), 2932 (s), 2874 (s), 1707 (s), 1668 (m), 1464 (s), 1448 (s), 1431 (s), 1386 (m), 1367 (m), 1321 (m), 1254 (m), 1225 (m), 1205 (m), 1129 (s), 1093 (m), 1045 (m), 998 (m), 939 (m), 897 (m), 832 (m), 810 (m).

EI-MS (70 eV): *m/z* [%] = 206 (100) [M]$^+$, 191 (42) [M-CH$_3$]$^+$, 178 (9), 164 (42), 163 (42), 145 (70), 136 (57), 121 (70), 107 (24), 94 (59), 79 (71), 69 (22), 55 (35), 41 (37).

(4S,4aR*,8aR*)-4-Isopropyl-6-methyl-3,4,4a,7,8,8a-hexahydronaphthalen-1(2*H*)-one (**11**)*

^{1}H-NMR (600 MHz, CDCl$_3$, TMS): δ [ppm] = 5.56-5.52 (m, 1 H), 2.39-2.35 (m, 2 H), 2.32-2.22 (m, 1 H), 2.10-2.01 (m, 1 H), 2.01-1.84 (m, 7 H), 1.62 (s, 3 H), 1.60-1.49 (m, 1 H), 1.00-0.96 (m, 3 H), 0.90- 0.87 (m, 3 H).

^{13}C{^{1}H}-NMR (151 MHz, CDCl$_3$, TMS): δ [ppm] = 213.1 (1 C, C$_q$), 135.9 (1 C, C$_q$), 121.5 (1 C, CH), 51.2 (1 C, CH), 46.1 (1 C, CH), 44.3 (1 C, CH), 41.1 (1 C, CH$_2$), 29.8 (1 C, CH$_2$), 26.4 (1 C, CH), 25.5 (1 C, CH$_3$), 23.9 (1 C, CH$_2$), 22.0 (1 C, CH$_2$), 21.7 (1 C, CH$_3$), 15.1 (1 C, CH$_3$).

IR (solid phase): ν max [cm^{-1}] = 3012 (m), 2960 (s), 2929 (s), 2871 (s), 2835 (s), 1715 (s), 1670 (m), 1453 (s), 1369 (s), 1316 (m), 1237 (m), 1206 (m), 1146 (m), 1065 (m), 1031 (w), 973 (w), 875 (w), 849 (m), 831 (w), 785 (w).

EI-MS (70 eV): *m/z* [%] = 206 (95) [M]$^+$, 191 (32) [M-CH$_3$]$^+$, 178 (12), 164 (45), 163 (40), 145 (73), 136 (61), 121 (100), 105 (27), 91 (49), 79 (70), 55 (42), 41 (42).

Isomerization of 9, 10, and 11

Ketones **9**, **10** and **11** (0.5 g, 2.43 mmol, 1.0 equiv) in methanol (10 mL) were mixed with NaOMe in methanol (5.4 M, 11.24 mL, 60.75 mmol, 25.0 equiv) at room temperature and stirred for 60 h. The reaction was terminated by the addition of sat. NH$_4$Cl solution (10 mL). The phases were separated and the aqueous phase was extracted five times with pentane (15 mL each). The combined organic phases were washed with NaCl solution (10 mL), dried over MgSO$_4$, and the solvent was removed. After column chromatography, 190 mg (0.92 mmol, 38%) of the ketone mixture was recovered, leading to a 1:3:3 mixture of **9**, **10** and **11**, that was used in the next step without further purification [S11].

Amorph-4-ene-10β-ol (1*S,4*S**,4a*S**,8a*R**)-4-isopropyl-1,6-dimethyl-1,2,3,4,4a,7,8,8a-octahydronaphthalen-1-ol (14)), δ-cadinol ((1*R**,4*S**,4a*R**,8a*S**)-4-isopropyl-1,6-dimethyl-1,2,3,4,4a,7,8,8a-octahydronaphthalen-1-ol (12)), and τ-cadinol (1*S**,4*S**,4a*R**,8a*R**)-4-isopropyl-1,6-dimethyl-1,2,3,4,4a,7,8,8a-octahydronaphthalen-1-ol (13))**

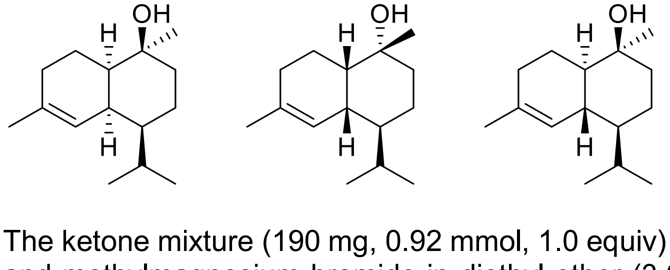

The ketone mixture (190 mg, 0.92 mmol, 1.0 equiv) was dissolved in dry diethyl ether (15 mL) and methylmagnesium bromide in diethyl ether (3.0 M, 0.46 mL, 1.38 mmol, 1.5 equiv) was added dropwise under ice cooling. After stirring the solution for 30 min at room temperature the reaction was terminated by the addition of sat. NH$_4$Cl solution and water (5 mL each). The phases were separated, and the aqueous phase was extracted three times with diethyl ether

(15 mL each). The combined organic phases were dried over $MgSO_4$. The solvent was removed and after column chromatographic purification [silica-gel, *n*-pentane/diethyl ether 2:1] three fractions were obtained. The product **12** (110 mg, 0.50 mmol, 54%) was obtained as a white solid. The product **13** (70 mg, 0.32 mmol, 35%) was obtained as a colorless liquid. Product **14** was obtained by reversed-phase column chromatography [LicroPrep RP-18 phase, MeOH/H_2O 10:1] as a clear liquid (19 mg, 0.086 mmol, 9%). The spectroscopic data obtained are in agreement with literature data [S11,S13,S14].

Amorph-4-ene-10β-ol (1S,4S*,4aS*,8aR*)-4-isopropyl-1,6-dimethyl-1,2,3,4,4a,7,8,8a-octahydronaphthalen-1-ol (**14**))*

I=1596 (HP-5-MS) (Lit.: 1592 (CPSil-5) [S14])

DC [silica-gel, *n*-pentane/diethyl ether (2:1)]: R_f = 0.80 [PMA = purple].

^{1}H-NMR (500 MHz, $CDCl_3$, TMS): δ [ppm] = 5.45-5.45 (m, 1 H), 2.65-2.58 (m, 1 H), 2.43-2.33 (m, 1 H), 2.22 (dddd, 1 H, ^3J = 14.0 Hz, ^3J = 7.7 Hz, ^3J = 2.4 Hz, ^3J = 1.2 Hz), 1.96-1.88 (m, 1 H), 1.74 (dddd, 1 H, ^3J = 13.7 Hz, ^3J = 11.0 Hz, ^3J = 8.3 Hz, ^3J = 5.2 Hz), 1.68-1.59 (m, 3 H), 1.67 (s, 3 H), 1.50-1.48 (m, 1 H), 1.33 (ddd, 1 H, ^3J = 15.8 Hz, ^3J = 12.4 Hz, ^3J = 3.2 Hz), 1.19 (s, 3 H), 1.15-0.94 (m, 2 H), 0.96 (d, 3 H, ^3J = 6.5 Hz), 0.88 (d, 3 H, ^3J = 6.6 Hz).

$^{13}C\{^1H\}$-NMR (151 MHz, $CDCl_3$, TMS): δ [ppm] = 140.0 (1 C, C_q), 120.7 (1 C, CH), 72.9 (1 C, C_q), 48.2 (1 C, CH), 41.9 (1 C, CH_2), 41.4 (1 C, CH), 36.0 (1 C, CH), 28.8 (1 C, CH_2), 28.7 (1 C, CH), 27.6 (1 C, CH), 23.9 (1 C, CH_2), 23.8 (1 C, CH_3), 22.2 (1 C, CH_2), 21.6 (1 C, CH_3), 20.5 (1 C, CH_3). ^{1}H- and ^{13}C-NMR data match those of Weyerstahl et al. [S14].

IR (solid phase): ν_{max} [cm^{-1}] = 3563 (br. s), 2922 (s), 2876 (s), 1642 (w), 1445 (m), 1379 (m), 1301 (m), 1265 (w), 1170 (m), 732 (m).

EI-MS (70 eV): *m/z* [%] = 222 ([M]$^+$, 2), 204 (42), 189 (11), 161 (100), 147 (7), 138 (14), 119 (67), 105 (71), 93 (46), 79 (32), 69 (13), 55 (19), 43 (42).

δ-Cadinol ((1R,4S*,4aR*,8aS*)-4-isopropyl-1,6-dimethyl-1,2,3,4,4a,7,8,8a-octahydronaphthalen-1-ol (**12**))*

I = 1645 (HP-5-MS)

DC [silica-gel, *n*-pentane/diethyl ether (2:1)]: R_f = 0.19 [PMA = purple].

^{1}H-NMR (600 MHz, $CDCl_3$, TMS): δ [ppm] = 5.53-5.48 (m, 1 H), 2.05-1.85 (m, 5 H), 1.65 (s, 3 H), 1.63-1.45 (m, 5 H), 1.35-1.24 (m, 1 H), 1.29 (s, 3 H), 1.15-1.06 (m, 1 H), 0.88 (d, 3 H, ^3J = 7.0 Hz), 0.81 (d, 3 H, ^3J = 7.0 Hz).

$^{13}C\{^1H\}$-NMR (151 MHz, $CDCl_3$, TMS): δ [ppm] = 134.5 (1 C, C_q), 124.8 (1 C, CH), 72.7 (1 C, C_q), 45.7 (1 C, CH), 44.2 (1 C, CH), 36.9 (1 C, CH), 35.5 (1 C, CH_2), 31.3 (1 C, CH_2), 28.1 (1 C, CH_3), 26.6 (1 C, CH), 23.8 (1 C, CH_3), 21.8 (1 C, CH_3), 21.7 (1 C, CH_2), 18.7 (1 C, CH_2), 15.5 (1 C, CH_3). ^{1}H- and ^{13}C-NMR data match those of Daub et al. [S13].

IR (solid phase): ν_{max} [cm^{-1}] = 3370 (s), 3012 (m), 2960 (s), 2936 (s), 2872 (s), 2830 (s), 1465 (s), 1374 (s), 1339 (m), 1295 (m), 1249 (m), 1234 (m), 1204 (m), 1154 (m), 1130 (s), 1110 (s), 1048 (s), 1015 (m), 987 (m), 950 (s), 924 (m), 888 (m), 874 (s), 829 (s), 772 (m).

EI-MS (70 eV): *m/z* [%] = 204 (32), 189 (9), 161 (100), 147 (5), 133 (9), 119 (49), 105 (38), 95 (26), 79 (22), 69 (10), 58 (11), 43 (28), 41 (15).

τ-Cadinol (1S,4S*,4aR*,8aR*)-4-isopropyl-1,6-dimethyl-1,2,3,4,4a,7,8,8a-octahydronaphthalen-1-ol (**13**)*

I=1639 (HP-5-MS)

DC [silica-gel, *n*-pentane/diethyl ether (2:1)]: R_f = 0.40 [PMA = purple].

^{1}H-NMR (600 MHz, $CDCl_3$, TMS): δ [ppm] = 5.54 (s, 1 H), 2.18 (dsept, 1 H, ^3J = 6.9 Hz, ^3J = 3.3 Hz,), 2.07-1.88 (m, 4 H), 1.76-1.70 (m, 1 H), 1.67-1.65 (m, 3 H), 1.50-1.30 (m, 4 H), 1.21 (s, 3 H), 1.12-1.04 (m, 1 H), 1.04-0.95 (m, 1 H), 0.91 (d, 3 H, ^3J = 6.8 Hz), 0.79 (d, 3 H, ^3J = 6.8 Hz).

^{13}C{^{1}H}-NMR (151 MHz, $CDCl_3$, TMS): δ [ppm] = 134.5 (1 C, C_q), 122.8 (1 C, CH), 70.8 (1 C, C_q), 48.1 (1 C, CH), 46.8 (1 C, CH), 40.5 (1 C, CH_2), 37.9 (1 C, CH), 31.1 (1 C, CH_2), 28.6 (1 C, CH_3), 26.3 (1 C, CH), 23.9 (1 C, CH_3), 22.7 (1 C, CH_2), 21.6 (1 C, CH_3), 20.0 (1C, CH_2), 15.3 (1 C, CH_3). ^{1}H- and ^{13}C-NMR data match those of Labbe et al. [S15].

IR (solid phase): ν_{max} [cm^{-1}] = 3407 (s), 3015 (m), 2959 (s), 2934 (s), 2971 (s), 2835 (s), 1465 (s), 1385 (s), 1298 (m), 1238 (m), 1193 (m), 1145 (s), 1095 (m), 1033 (m), 1012 (m), 990 (m), 906 (s), 880 (m), 771 (m).

EI-MS (70 eV): *m*/*z* [%] = 222 ($[M]^+$, <1), 204 (44) $[M-18]^+$, 189 (17), 161 (100), 147 (3), 134 (17), 119 (18), 105 (26), 95 (16), 81 (19), 69 (6), 55 (7), 43 (19), 41 (11).

Enantioselective synthesis

(*S*)-2-Isopropyl-5-oxohexanal (*S*-17)

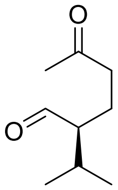

In a manner similar to Chen et al. [S16,S17] freshly distilled isovaleraldehyde (**1**, 2.69 mL, 2.15 g, 25.0 mmol, 1.0 equiv), (*S*)-(−)-2-(1-methoxy-1,1-diphenylmethyl)pyrrolidine ((*S*)-Jørgensen's organocatalyst [S18], *S*-**16**, 0.407 mg, 1.25 mmol, 0.05 equiv) and cocatalyst ethyl 3,4-dihydroxybenzoate (0.911 g, 5.0 mmol, 0.2 equiv) were added to a Schlenk tube (see Figure S4) and cooled to 0 °C. Then, freshly distilled methyl vinyl ketone (**15**, 3.13 mL, 2.63 g, 37.5 mmol, 1.5 equiv) was added and the mixture stirred for 36 h at 4 °C under nitrogen. To keep the low temperature for 36 h the tube was placed in a LAUDA Proline cryostat (see Figure S4). The crude mixture was purified by column chromatography [silica-gel, *n*-pentane/diethyl ether 1:1]. The product (*S*-**17**, 3.41 g, 21.83 mmol, 87%) was obtained as slightly yellow liquid. The enantiomeric excess was determined by chiral GC to be 99%.

DC [silica-gel, *n*-pentane/diethyl ether 1:1]: R_f = 0.56 [*p*-anisaldehyde/H_2SO_4 = purple].

Chiral GC [Hydrodex β-6TBDM 50-5-25-120-10-230]: *ee* = 99%.

^{1}H-NMR} (400 MHz, $CDCl_3$, TMS): δ [ppm] = 9.61 (d, ^3J(H,H)=2.8 Hz, 1H, CHO), 2.55 - 2.47 (m, 1H, CH), 2.42 - 2.34 (m, 1H, CH), 2.13 (s, 3H, CH_3), 2.11 - 2.01 (m, 2H, CH_2), 1.89 - 1.72 (m, 2H, CH_2), 1.00 (d, ^3J(H,H)=6.7 Hz, 3H, CH_3), 0.97 (d, ^3J(H,H)=6.7 Hz, 3H, CH_3).

$^{13}C\{^1H\}$-NMR (100 MHz, $CDCl_3$, TMS): δ [ppm] = 207.9 (CO), 205.1 (CHO), 57.4 (CH), 41.1 (CH_2), 29.8 (CH_3), 28.2 (CH), 20.1 (CH_3), 19.3 (CH_3), 19.2 (CH_2).

IR (solid phase): ν_{max} [cm^{-1}] = 2963 (s), 2876 (m), 2827 (w), 2723 (w), 1718 (s), 1468 (w), 1412 (w), 1371 (m), 1235 (w), 1170 (m), 865 (w).

EI-MS (70 eV): *m/z* [%] = 123 (11), 110 (18), 95 (24), 86 (10), 85 (14), 81 (10), 71 (31), 70 (14), 69 (24), 59 (11), 58 (86), 55 (20), 43 (100), 42 (8), 41 (35), 39 (19).

$[\alpha]_D^{27}$ = +2.53 (c = 1.0, $CHCl_3$).

(*R*)-2-Isopropyl-5-oxohexanal (*R*-17)

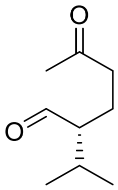

The synthesis of the *R*-enantiomer was performed identical to that of the *S*-enantiomer, using (*R*)-(+)-2-(1-methoxy-1,1-diphenylmethyl)pyrrolidine ((*R*)-Jørgensen's organo-catalyst *R*-**16**) [S18] catalyst. The product *R*-**17** (3.41 g, 21.83 mmol, 87%) was obtained as a slight yellow liquid with an enantiomeric excess of 98% as determined by chiral GC.

DC [silica-gel, *n*-pentane/diethyl ether (1:1)]: R_f = 0.56 [*p*-anisaldehyde/H_2SO_4 = purple].

Chiral GC [Hydrodex β-6TBDM 50-5-25-120-10-230]: *ee* = 98%.

^1H-NMR (400 MHz, $CDCl_3$, TMS): δ [ppm] = 9.62 (d, 1H, ^3J = 2.8 Hz, CHO), 2.55 - 2.47 (m, 1H, CH), 2.41 - 2.33 (m, 1H, CH), 2.13 (s, 3H, CH_3), 2.20 – 1.96 (m, 2H, CH_2), 1.92 – 1.69 (m, 2H, CH_2), 1.00 (d, ^3J = 6.6 Hz, 3H, CH_3), 0.97 (d, ^3J= 6.6 Hz, 3H, CH_3).

$^{13}C\{^1H\}$-NMR (100 MHz, $CDCl_3$, TMS): δ [ppm] = 208.0 (CO), 205.2 (CHO), 57.5 (CH), 41.2 (CH_2), 30.0 (CH_3), 28.3 (CH), 20.2 (CH_3), 19.5 (CH_3), 19.3 (CH_2).

IR (solid phase): ν_{max} [cm^{-1}] = 2963 (s), 2876 (m), 2827 (w), 2723 (w), 1718 (s), 1468 (w), 1412 (w), 1371 (m), 1235 (w), 1170 (m), 865 (w).

EI-MS (70 eV): *m/z* [%] = 123 (6), 110 (10), 95 (16), 86 (7), 85 (10), 81 (7), 71 (26), 70 (12), 69 (21), 59 (10), 58 (83), 55 (18), 43 (100), 42 (7), 41 (32), 39 (15).

$[\alpha]_D^{25}$ = -2.82 (c = 1.0, $CHCl_3$).

(*S,E*)-5-Isopropyl-8-methylnona-6,8-dien-2-one (*S*-18)

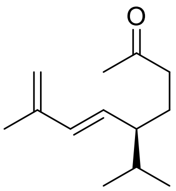

In a manner analogous to Nishikawa et al. [S9], diethyl (2-methylallyl)phosphonate (4.610 g, 24.0 mmol, 1.5 equiv) was dissolved in 60 mL dry THF, cooled to −78 °C and stirred for 10 minutes. Then, *n*-BuLi in hexane (1.6 M, 6.5 mL, 10.4 mmol, 1.6 equiv) was added dropwise at −78 °C. The mixture was stirred for 45 min before the aldehyde *S*-**17** (1.015 g, 6.5 mmol, 1.0 equiv) was added. After 10 minutes, the reaction mixture was allowed to very slowly warm to rt and stirred for 8 h. The reaction was quenched by the addition of sat. NH_4Cl solution, the organic layer was separated and the aqueous layer was extracted with diethyl ether. The combined organic phases were dried over $MgSO_4$, the solvent was removed under reduced pressure and the product was purified by column chromatography [silica-gel, *n*-pentane/diethyl ether 5:1]. Compound *S*-**18** (0.631 g, 3.25 mmol, 51%) was obtained as colorless liquid.

DC [silica-gel, *n*-pentane/diethyl ether 5:1]: R_f = 0.63 [*p*-anisaldehyde/H_2SO_4 = purple].

^{1}H-NMR (400 MHz, $CDCl_3$, TMS): δ [ppm] = 6.06 (d, ^3J = 15.6 Hz, 1H, CH), 5.35 (dd, ^3J = 15.7 Hz, ^3J = 9.4 Hz, 1H, CH), 4.89 - 4.86 (m, 2H, CH_2), 2.46 - 2.28 (m, 2H, CH_2), 2.11 (s, 3H, CH_3), 1.86 - 1.82 (m, 3H, CH_3), 1.81 - 1.73 (m, 2H, CH_2), 1.66 - 1.58 (m, 1H, CH), 1.54 - 1.46 (m, 1H, CH), 0.89 (d, ^3J = 6.7 Hz, 3H, CH_3), 0.84 (d, ^3J = 6.8 Hz, 3H, CH_3).

^{13}C{^{1}H}-NMR (100 MHz, $CDCl_3$, TMS): δ [ppm] = 209.4 (CO), 142.0 (C_q), 134.4 (CH), 132.2 (CH), 114.6 (CH_2), 49.4 (CH), 42.1 (CH_2), 32.4 (CH), 30.1 (CH_3), 26.3 (CH_2), 20.7 (CH_3), 19.2 (CH_3), 18.8 (CH_3).

IR (solid phase): ν_{max} [cm^{-1}] = 3081 (w), 2960 (s), 2873 (m), 1717 (s), 1610 (w), 1456 (w), 1436 (w), 1367 (m), 1311 (w), 1254 (w), 1164 (m), 972 (m), 885 (m).

EI-MS (70 eV): *m*/*z* [%] = 194 (11), 136 (40), 123 (8), 121 (48), 107 (9), 95 (9), 94 (13), 93 (100), 91 (25), 81 (15), 79 (23), 77 (23), 43 (58), 41 (18), 39 (10).

$[\alpha]_D^{28}$ = +1.99 (c = 1.0, $CHCl_3$).

(*R,E*)-5-Isopropyl-8-methylnona-6,8-dien-2-one (*R*-18)

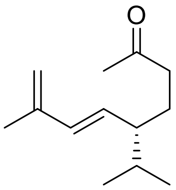

The synthesis of the *R*-enantiomer was performed identical to that of the *S*-enantiomer using *R*-**17**and diethyl (2-methylallyl)phosphonate. The product *R*-**18** (0.640 g, 3.25 mmol, 50%) was obtained after column chromatography [silica-gel, *n*-pentane/diethyl ether 5:1] as a clear colorless liquid.

$[\alpha]_D^{28}$ = --2.53 (c = 1.0, $CHCl_3$).

Diisopropylammonium 2,2,2-trifluoracetate (DIA TFA)

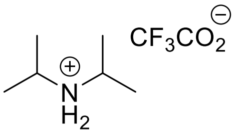

By the method of Bugarin et al. [S19] TFA (7.70 mL, 11.40 g, 100.00 mmol, 1.00 equiv) was added dropwise to a stirred solution of diisopropylamine (14.05 mL, 10.12 g, 100.00 mmol, 1.00 equiv) in diethyl ether (100 mL) at 0 °C. The reaction mixture was stirred for further 5 min at 0 °C. The formed crystals were filtered, washed with diethyl ether and dried in vacuum. The product was obtained as white crystalline powder in a quantitative yield.

^{1}H-NMR (400 MHz, $CDCl_3$, TMS): δ [ppm] = 8.96 (s, 1H, COOH), 3.38 (dh, ^3J = 13.0 Hz, ^3J = 6.5 Hz, 2H, CH), 1.33 (d, ^3J = 6.5 Hz, 12 H, CH_3).

^{13}C{^{1}H}-NMR (100 MHz, $CDCl_3$, TMS): δ [ppm] = 161.9 (COOH), 118.1 (CF_3), 46.9 (CH), 18.8 (CH_3).

^{19}F{^{1}H}-NMR (377 MHz, $CDCl_3$, $CFCl_3$): δ [ppm] = -76.3 (CF_3).

IR (ATR, Diamond): ν_{max} [cm^{-1}] = 3048 (w), 2984 (w), 2882 (w), 2731 (w), 2500 (w), 1669 (s), 1478 (w), 1429 (w), 1393 (w), 1319 (w), 1166 (s), 1119 (s), 948 (w), 827 (m), 813 (m), 714 (m).

(4*S*,4a*S*,8a*R*)-4-Isopropyl-6-methyl-3,4,4a,7,8,8a-hexahydronaphthalen-1(2*H*)-one (*S*-9), (4*S*,4a*R*,8a*S*)-4-isopropyl-6-methyl-3,4,4a,7,8,8a-hexahydronaphthalen-1(2*H*)-one (*S*-10) and (4*S*,4a*R*,8a*R*)-4-isopropyl-6-methyl-3,4,4a,7,8,8a-hexahydronaphthalen-1(2*H*)-one (*S*-11)

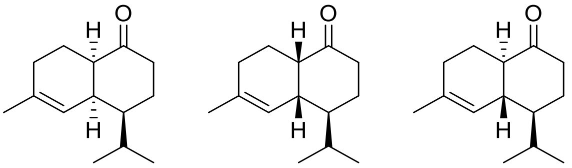

The reaction was carried out without a nitrogen atmosphere in a 5 mL round-bottomed flask equipped with a stirring bar. To diene *R*-**18** (0.253 g, 1.3 mmol, 1.0 equiv) dissolved in 2 mL THF were added formaldehyde in methanol (37%, 14 µL, 16 mg, 0.52 mmol, 0.4 equiv), DIA TFA (112 mg, 0.52 mmol, 0.4 equiv), and TFA (10 µL, 15 mg, 0.13 mmol, 0.1 equiv). The reaction mixture was stirred under reflux for 48 h (product formation monitored by GC–MS). After 48 h, maleic anhydride (0.153 g, 1.56 mmol, 1.2 equiv) was added to get rid of unreacted starting material (same R_f value of starting material and product). The intermolecular Diels–Alder reaction was finished after an additional 16 h. An inseparable mixture of the three diastereomers *S*-**9**, *S*-**10**, and *S*-**11** was formed and purified by column chromatography [silica-gel, *n*-pentane/diethyl ether 10:1]. The product containing the three ketones (55 mg, 0.267 mmol, 21%) was obtained as colorless oil. The analytical data are identical to the data reported for the racemic synthesis above.

DC [silica-gel, *n*-pentane/diethyl ether (10:1)]: R_f = 0.40-0.31 [*p*-anisaldehyde/H_2SO_4 = purple].

The spectroscopic data were identical to those of the racemic compounds, see above.

(4*R*,4a*R*,8a*S*)-4-Isopropyl-6-methyl-3,4,4a,7,8,8a-hexahydronaphthalen-1(2*H*)-one (*R*-9), (4*R*,4a*S*,8a*R*)-4-isopropyl-6-methyl-3,4,4a,7,8,8a-hexahydronaphthalen-1(2*H*)-one (*R*-10), and (4*R*,4a*S*,8a*S*)-4-isopropyl-6-methyl-3,4,4a,7,8,8a-hexahydronaphthalen-1(2*H*)-one (*R*-11)

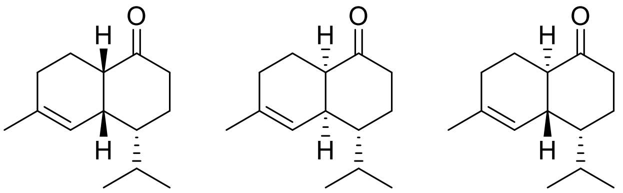

The respective 4*R*-enantiomers were obtained as described for the 4*S*-eantiomers. The product containing the three different diastereomers *R*-**9**, *R*-**10**, *R*-**11** (54 mg, 0.262 mmol, 20%) was purified by column chromatography [silica-gel, *n*-pentane/diethyl ether 10:1].

δ-Cadinol ((1*R*,4*S*,4a*R*,8a*S*)-4-isopropyl-1,6-dimethyl-1,2,3,4,4a,7,8,8a-octahydronaphthalen-1-ol, *S*-12), amorph-4-ene-10β-ol (1*S*,4*S*,4a*S*,8a*R*)-4-isopropyl-1,6-dimethyl-1,2,3,4,4a,7,8,8a-octahydronaphthalen-1-ol, *S*-14)) and τ-cadinol (1*S*,4*S*,4a*R*,8a*R*)-4-isopropyl-1,6-dimethyl-1,2,3,4,4a,7,8,8a-octahydronaphthalen-1-ol, *S*-13)

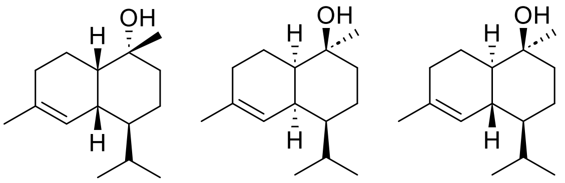

***S*-12** ***S*-14** ***S*-13**

The alcohols were synthesized as described above for the racemic synthesis of **12**, **13**, and **14**, starting from the respective 4*S*-ketones The crude product (22 mg, 0.107 mmol, 56%) was purified by two time column chromatography.

*(−)-δ-Cadinol ((1*R*,4*S*,4a*R*,8a*S*)-4-isopropyl-1,6-dimethyl-1,2,3,4,4a,7,8,8a-octahydronaphthalen-1-ol, S-**12**)*

RI (Kovats) = 1732 (Hydrodex-6-TBDM)

I (van den Dool) = 1727 (Hydrodex-6-TBDM)
Analytical data according to the racemic synthesis of δ-Cadinol **12**.

*Amorph-4-ene-10β-ol ((1*S*,4*S*,4a*S*,8a*R*)-4-isopropyl-1,6-dimethyl-1,2,3,4,4a,7,8,8a-octahydronaphthalen-1-ol, S-**14**)*

RI (Kovats) = 1623 (Hydrodex-6-TBDM)

I (van den Dool) = 1619 (Hydrodex-6-TBDM)

Analytical data according to the racemic synthesis of amorph-4-ene-10β-ol (**14**).

*τ-Cadinol ((1S,4S,4aR,8aR)-4-isopropyl-1,6-dimethyl-1,2,3,4,4a,7,8,8a-octahydronaphthalen-1-ol, S-**13**)*

RI (Kovats) = 1796 (Hydrodex-6-TBDM)

I (van den Dool) = 1795 (Hydrodex-6-TBDM)

Analytical data according to the racemic synthesis of τ-Cadinol **13**.

δ-Cadinol (1*S*,4*R*,4a*S*,8a*R*)-4-isopropyl-1,6-dimethyl-1,2,3,4,4a,7,8,8a-octahydronaphthalen-1-ol (*R*-12), amorph-4-ene-10β-ol (1*R*,4*R*,4a*R*,8a*S*)-4-isopropyl-1,6-dimethyl-1,2,3,4,4a,7,8,8a-octahydronaphthalen-1-ol (*R*-14), and τ-cadinol (1*R*,4*R*,4a*S*,8a*S*)-4-isopropyl-1,6-dimethyl-1,2,3,4,4a,7,8,8a-octahydronaphthalen-1-ol (*R*-13)

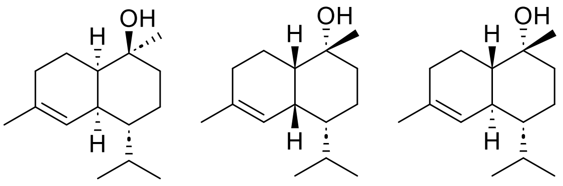

***R*-12** ***R*-14** ***R*-13**

The alcohols were synthesized as described above for within the racemic synthesis of **12**, **13**, and **14**, starting from the respective 4*R*-ketones The crude product (24 mg, 0.108 mmol, 58%) was purified by two time column chromatography.

*(+)-δ-Cadinol ((1S,4R,4aS,8aR)-4-isopropyl-1,6-dimethyl-1,2,3,4,4a,7,8,8a-octahydronaphthalen-1-ol, R-**12**)*

RI (Kovats) = 1738 (Hydrodex-6-TBDM)

I (van den Dool) = 1732 (Hydrodex-6-TBDM)

*Amorph-4-ene-10β-ol ((1R,4R,4aR,8aS)-4-Isopropyl-1,6-dimethyl-1,2,3,4,4a,7,8,8a-octahydronaphthalen-1-ol, R-**14**)*

RI (Kovats) = 1625 (Hydrodex-6-TBDM)

RI (Dool) = 1621 (Hydrodex-6-TBDM)

*(+)-τ-Cadinol ((1R,4R,4aS,8aS)-4-isopropyl-1,6-dimethyl-1,2,3,4,4a,7,8,8a-octahydronaphthalen-1-ol, R-**13**)*

RI (Kovats) = 1770 (Hydrodex-6-TBDM)

I (van den Dool) = 1765 (Hydrodex-6-TBDM)

Special reaction setups

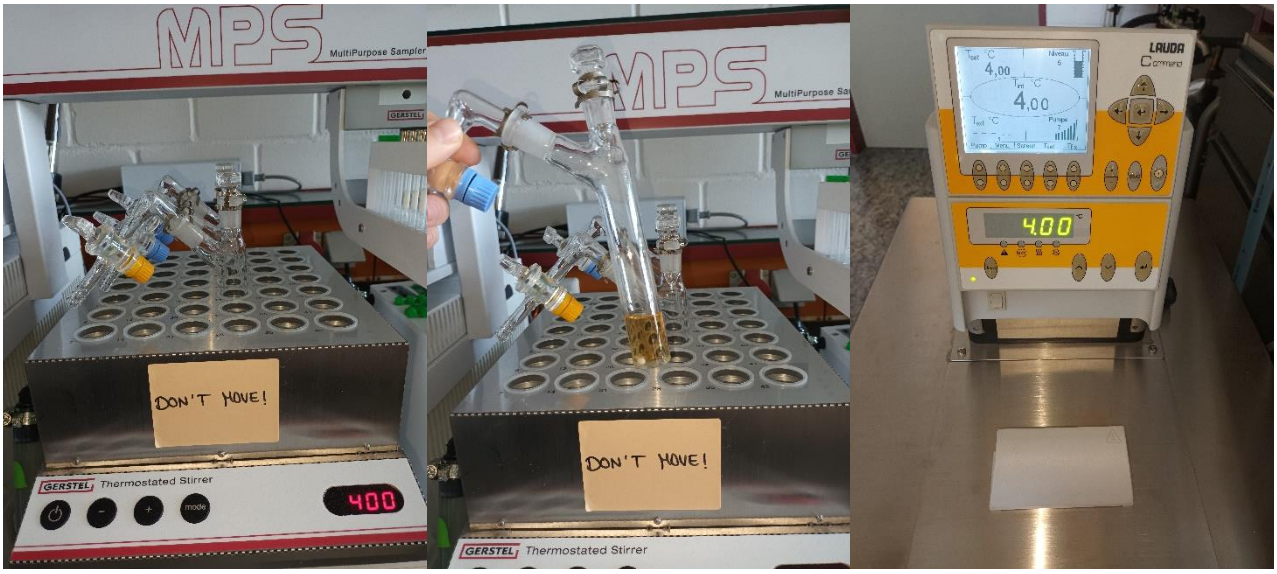

Figure S4: Michael addition (used for compounds *S*-**17** and *R*-**17**). The Michael addition was performed in a self-made tube (shown in the middle). The similar to a Schlenk reaction vessel fitted into the LAUDA Proline RP855 refrigerated thermostat, which was able to hold the temperature at a constant temperature of 4 °C for at least 36 h. Photo by A. Ladwig, not used previously elsewhere.

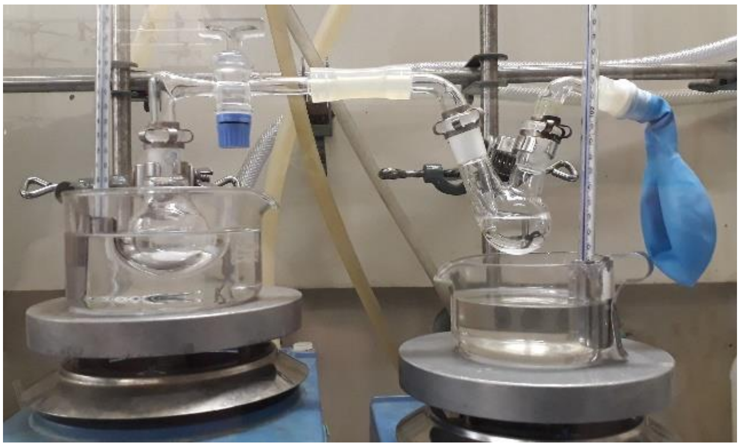

Figure S5: Cracking of paraformaldehyde (left flask) into formaldehyde. The left flask contains solid paraformaldehyde, which is thermally splitted. The formaldehyde produced is transferred into the solution of the right flask via the connection between tube and inlet. The balloon serves as monitor for the formaldehyde gas production. Photo by A. Ladwig, not used previously elsewhere.

References

S1 Ohta, Y.; Hirose, Y. *Bull. Chem. Soc. Jpn.* **1973,** *46*, 1535–1539.

S2 . Nagahama, S.; Tazaki, M.; Nomura, H.; Nishimura, K.; Tajima, M.; Iwasita, Y. *Mokuzai Gakkaishi* **1996,** *42*, 1127–1133.

S3 van den Dool, H.; Kratz, P. *J. Chromatogr.* **1963,** *11*, 463–471.

S4 . Kováts, E. *Helv. Chim. Acta* **1958,** *41*, 1915–1932.

S5 Menke, M.; Peram, P. S.; Starnberger, I.; Hödl, W.; Jongsma, G. F. M.; Blackburn, D. C.; Rödel, M.-O.; Vences, M.; Schulz, S. *Beilstein J. Org. Chem.* **2016,** *12*, 2731–2738.

S6 Storck, G.; Brizzolara, A.; Landesman, H.; Szmuszkovicz, J.; Terrell, R. *J. Am. Chem. Soc.* **1963,** *85*, 207–222.

S7 Pettit, G. R.; Fessler, D. C.; Paull, K. D.; Hofer, P.; Knight, J. C. *J. Org. Chem.* **1970,** *35*, 1398–1404.

S8 Amat, M.; Guignard, G.; Llor, N.; Bosch, J. *J. Org. Chem.* **2014,** *79*, 2792–2802.

S9 Nishikawa, K.; Nakahara, H.; Shirokura, Y.; Nogata, Y.; Yoshimura, E.; Umezawa, T.; Okino, T.; Matsuda, F. *J. Org. Chem.* **2011,** *76*, 6558–6573.

S10 Searles, S.; Kenneth, J.; Lutz, E. F. *J. Am. Chem. Soc.* **1957,** *79*, 948–951.

S11 Taber, D. F.; Gunn, B. P. *J. Am. Chem. Soc.* **1979,** *101*, 3992–3993.

S12 Tojo, G.; Fernandez, M. *Oxidation of Alcohols to Aldehydes and Ketones;* Springer: New York, 2006.

S13 Daub, M. E.; Prudhomme, J.; Ben Mamoun, C.; Le Roch, K. G.; Vanderwal, C. D. *ACS Med. Chem. Lett.* **2017,** *8*, 355–360.

S14 Weyerstahl, P.; Marschall, H.; Son, P. T.; Giang, P. M. *Flavour Fragr. J.* **1999,** *14*, 219–224.

S15 Labbe, C.; Castillo, M.; Connolly, J. D. *Phytochemistry* **1993,** *34*, 441–444.

S16 Chen, K.; Baran, P. S. *Nature* **2009,** *459*, 824–828.

S17 Chen, K.; Ishihara, Y.; Galán, M. M.; Baran, P. S. *Tetrahedron* **2010,** *66*, 4738–4744.

S18 Enders, D.; Kipphardt, H.; Gerdes, P.; Breña-Valle, L. J.; Bhushan, V. *Bull. Soc. Chim. Belges* **1988,** *97*, 691–704.

S19 Bugarin, A.; Jones, K. D.; Connell, B. T. *Chem. Commun.* **2010,** *46*, 1715–1717.

Spectral NMR data (^{1}H and ^{13}C NMR for compounds 12 and 14)

Amorph-4-ene-10β-ol ((1*S**,4*S**,4a*S**,8a*R**)-4-isopropyl-1,6-dimethyl-1,2,3,4,4a,7,8,8a-octahydronaphthalen-1-ol, 14))

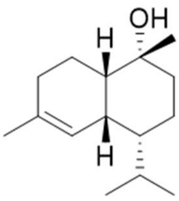

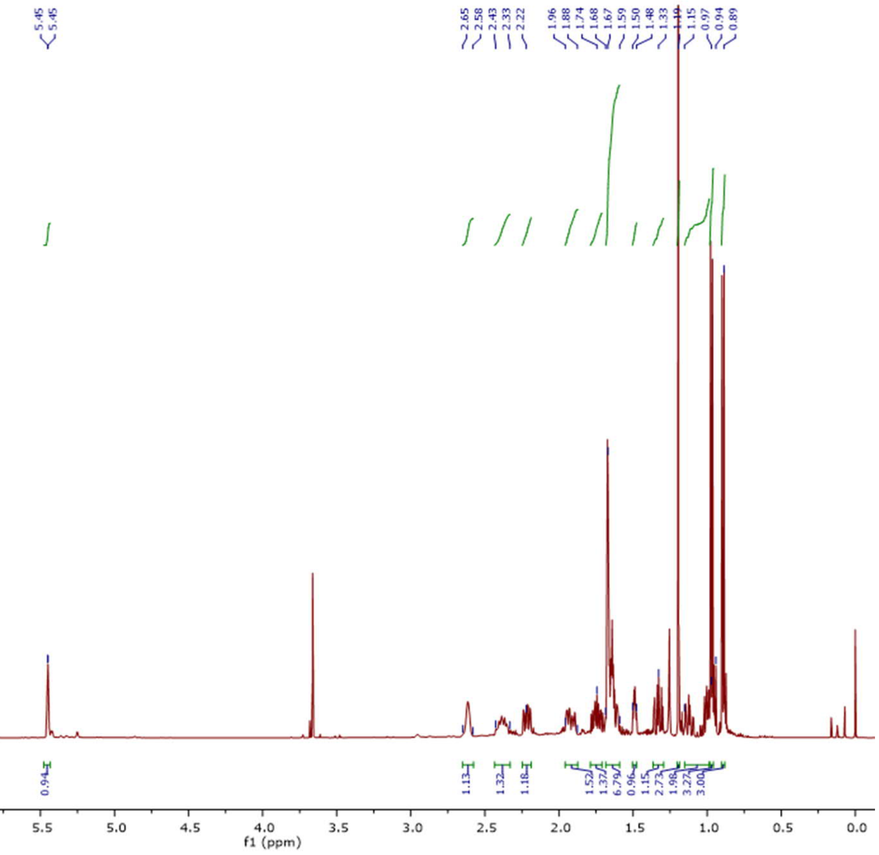

Figure S6: ^{1}H NMR (500 MHz) spectrum of amorph-4-ene-10β-ol (**14**).

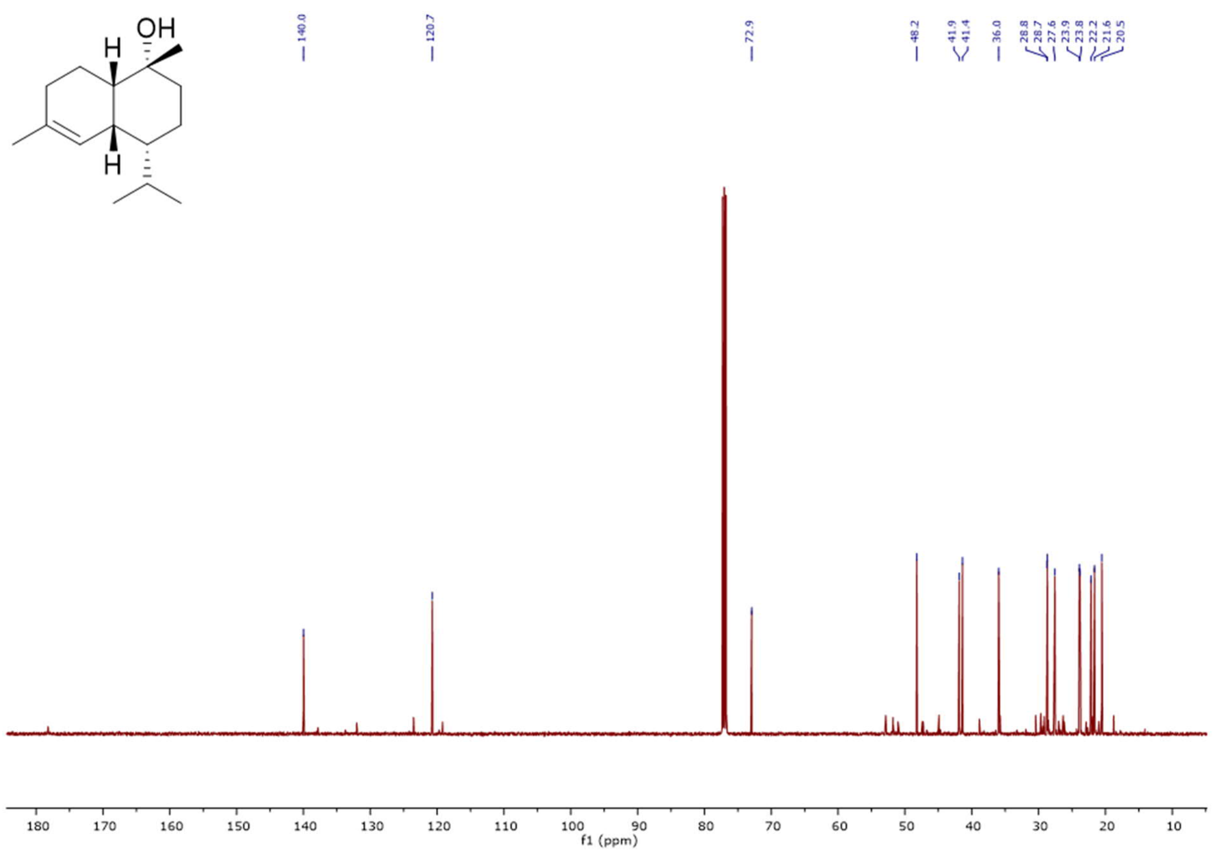

Figure S7: ^{13}C NMR (125 MHz) spectrum of amorph-4-ene-10β-ol (**14**).

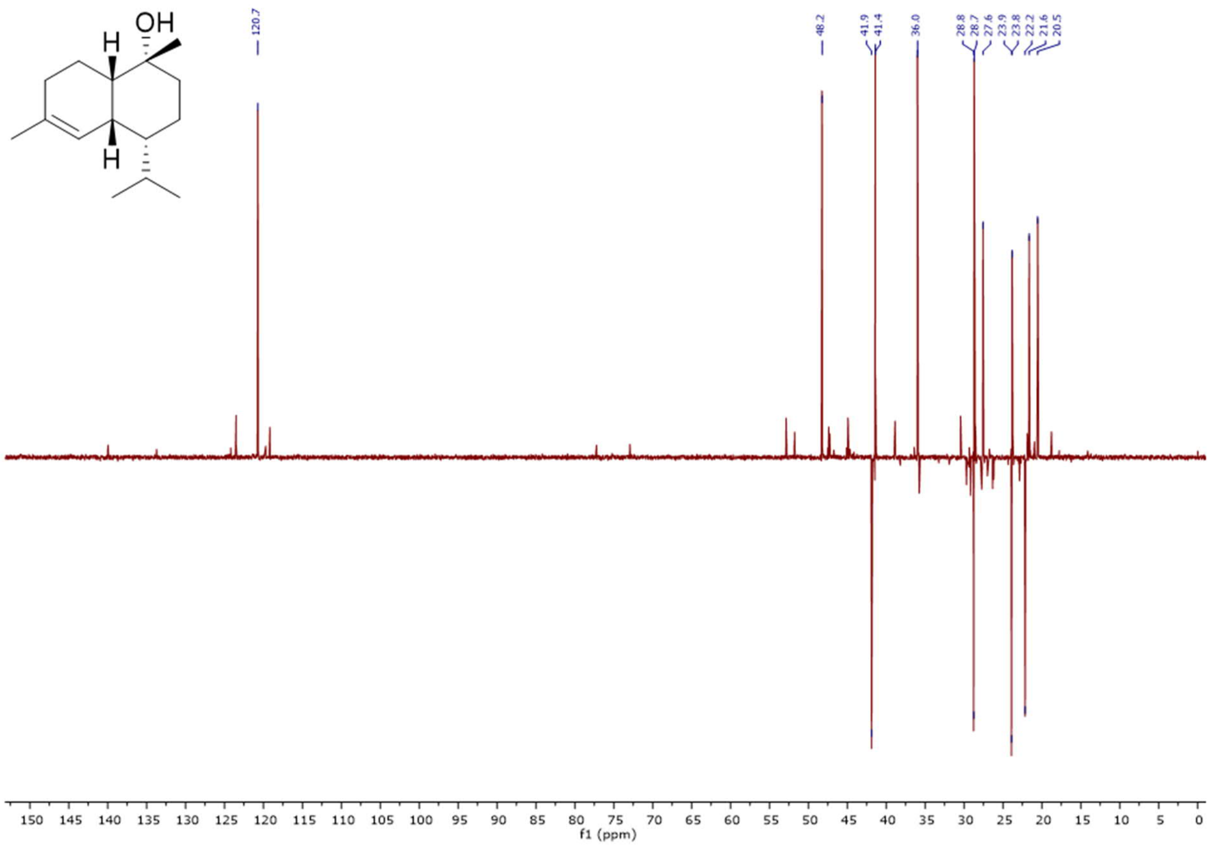

Figure S8: DEPT spectrum of amorph-4-ene-10β-ol (**14**).

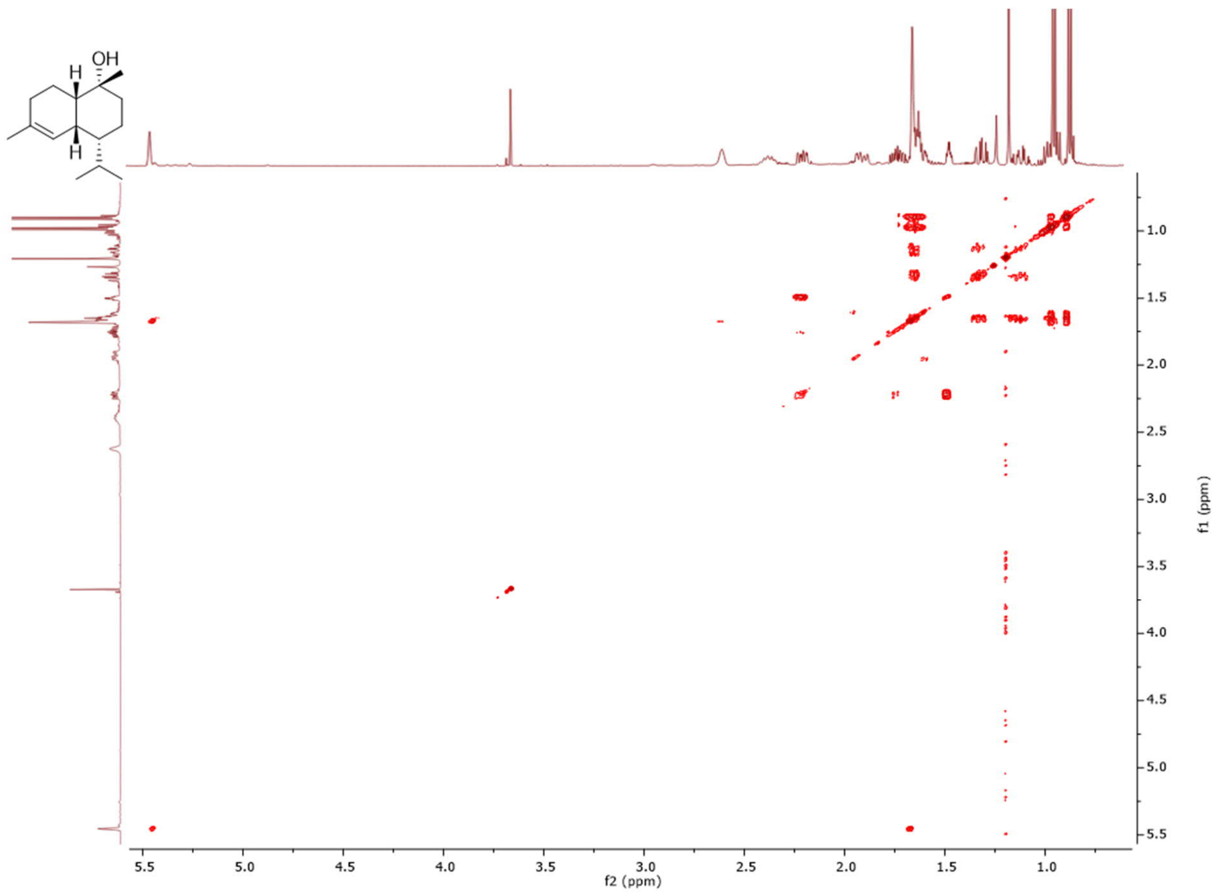

Figure S9: COSY spectrum of amorph-4-ene-10β-ol (**14**).

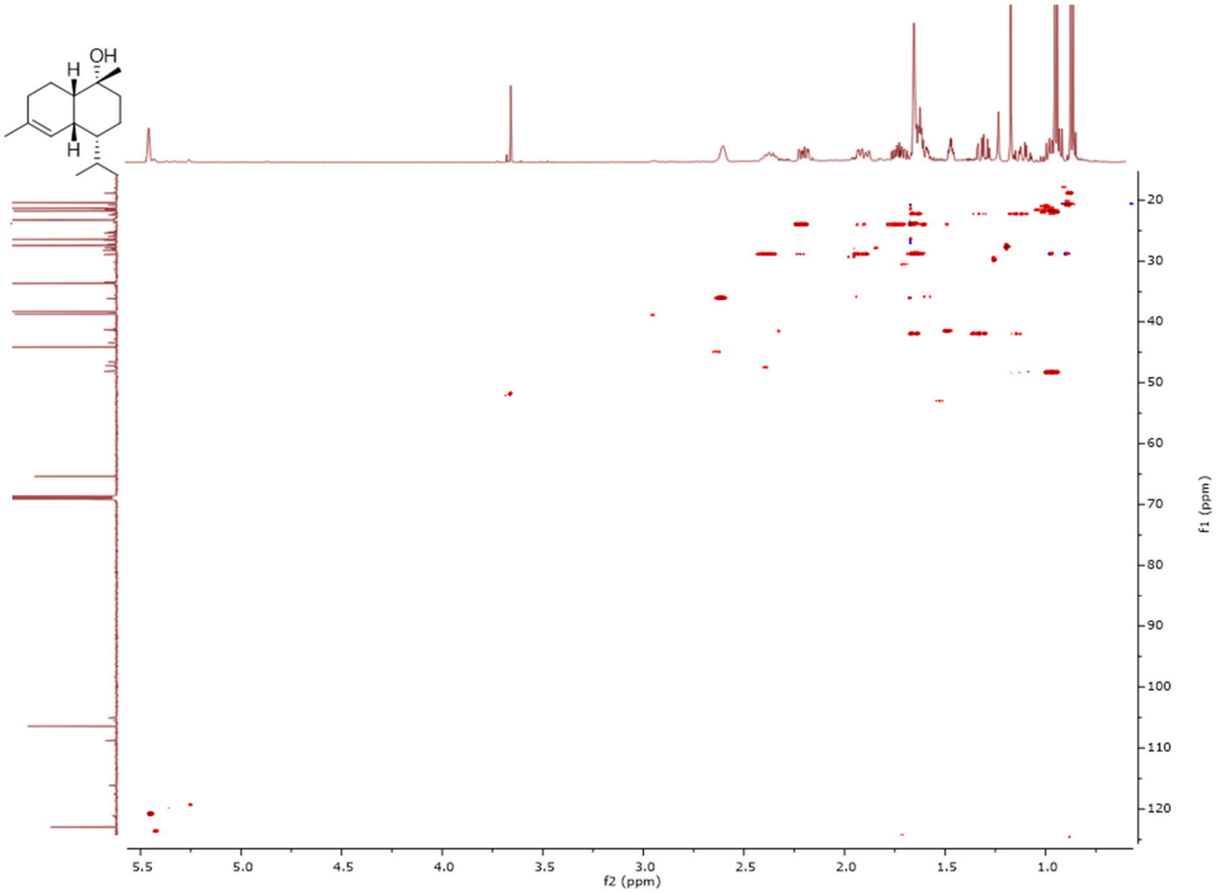

Figure S10: HSQC spectrum of amorph-4-ene-10β-ol (**14**).

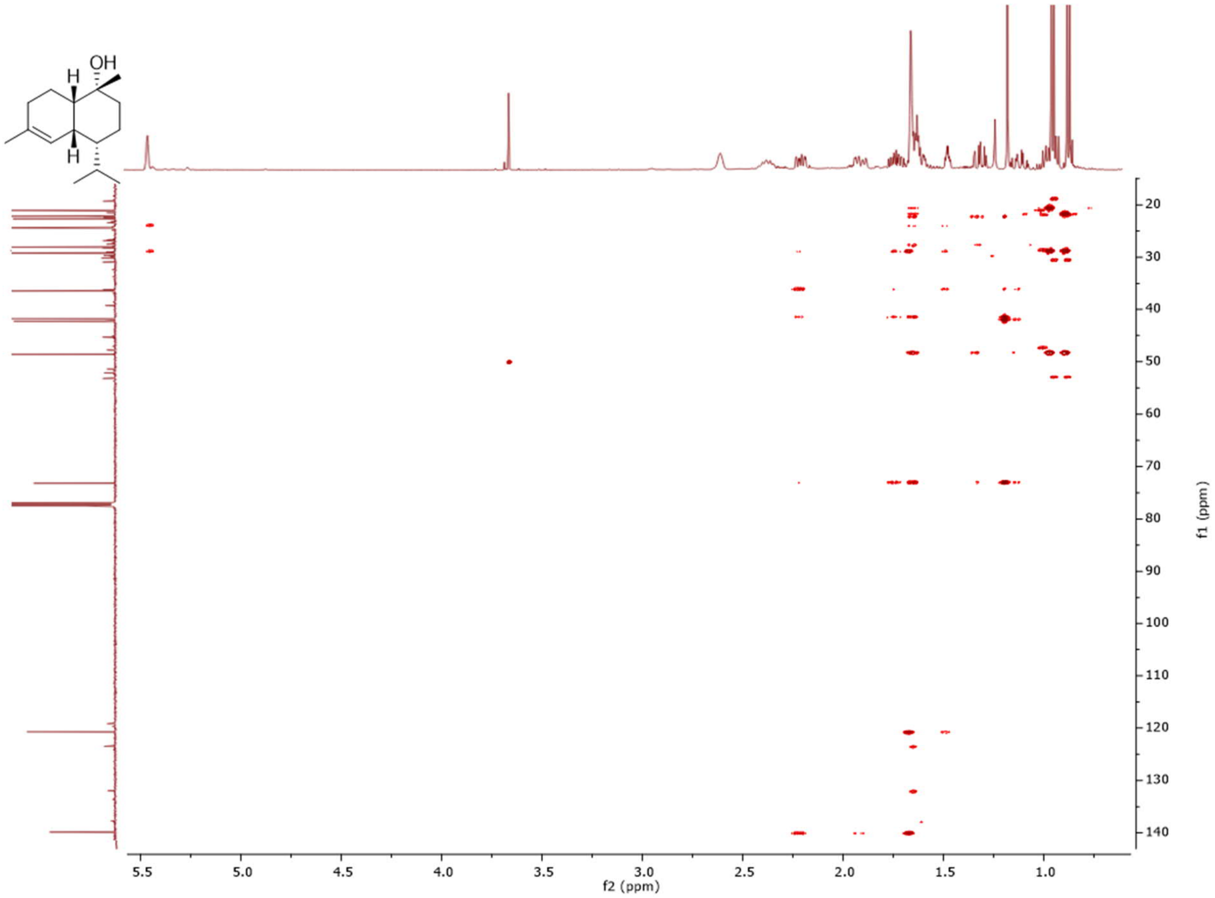

Figure S11: HMBC spectrum of amorph-4-ene-10β-ol (**14**).

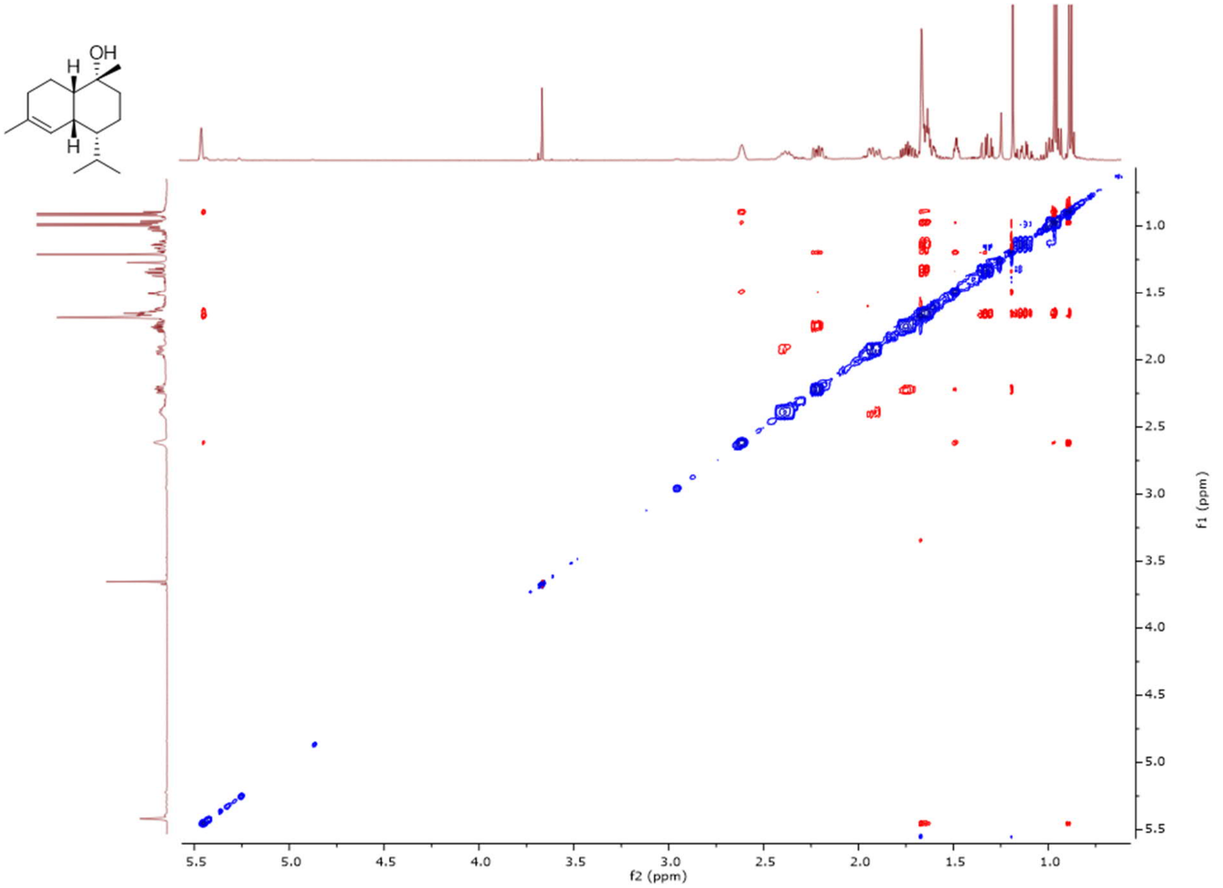

Figure S12: NOESY spectrum of amorph-4-ene-10β-ol (**14**).

δ-Cadinol (1*S,4*R**,4a*S**,8a*R**)-4-Isopropyl-1,6-dimethyl-1,2,3,4,4a,7,8,8a-octahydronaphthalen-1-ol, 12)**

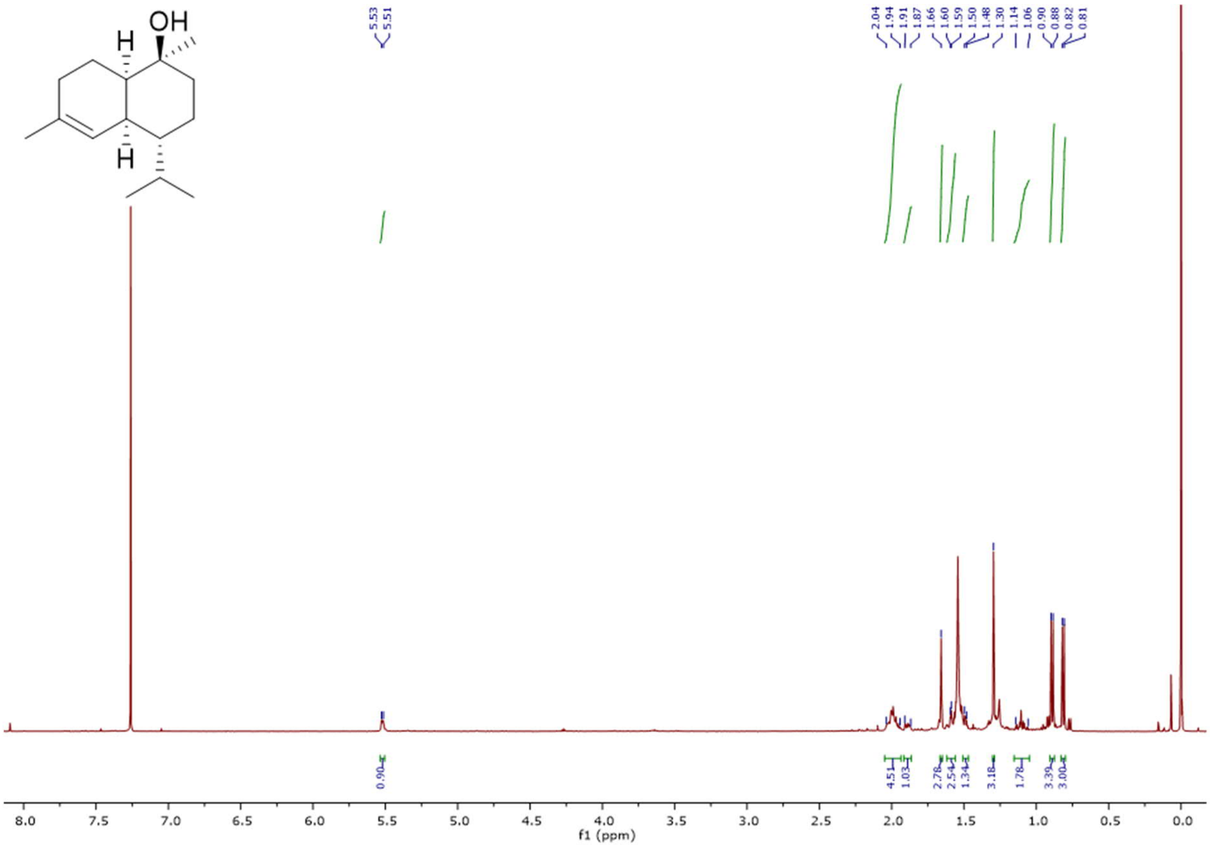

Figure S13: ^{1}H NMR (600 MHz) spectrum of δ-cadinol (**12**).

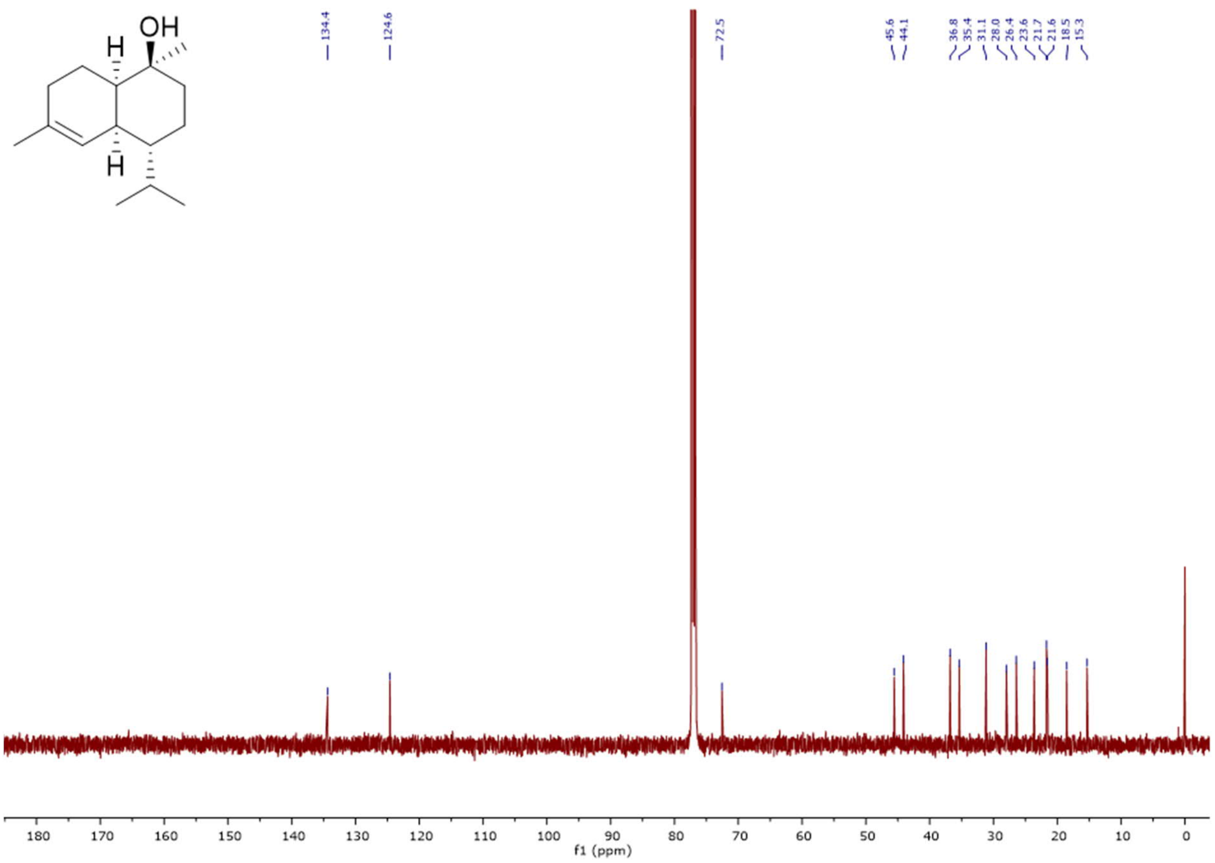

Figure S14: ^{13}C NMR (151 MHz) spectrum of δ-cadinol (**12**).

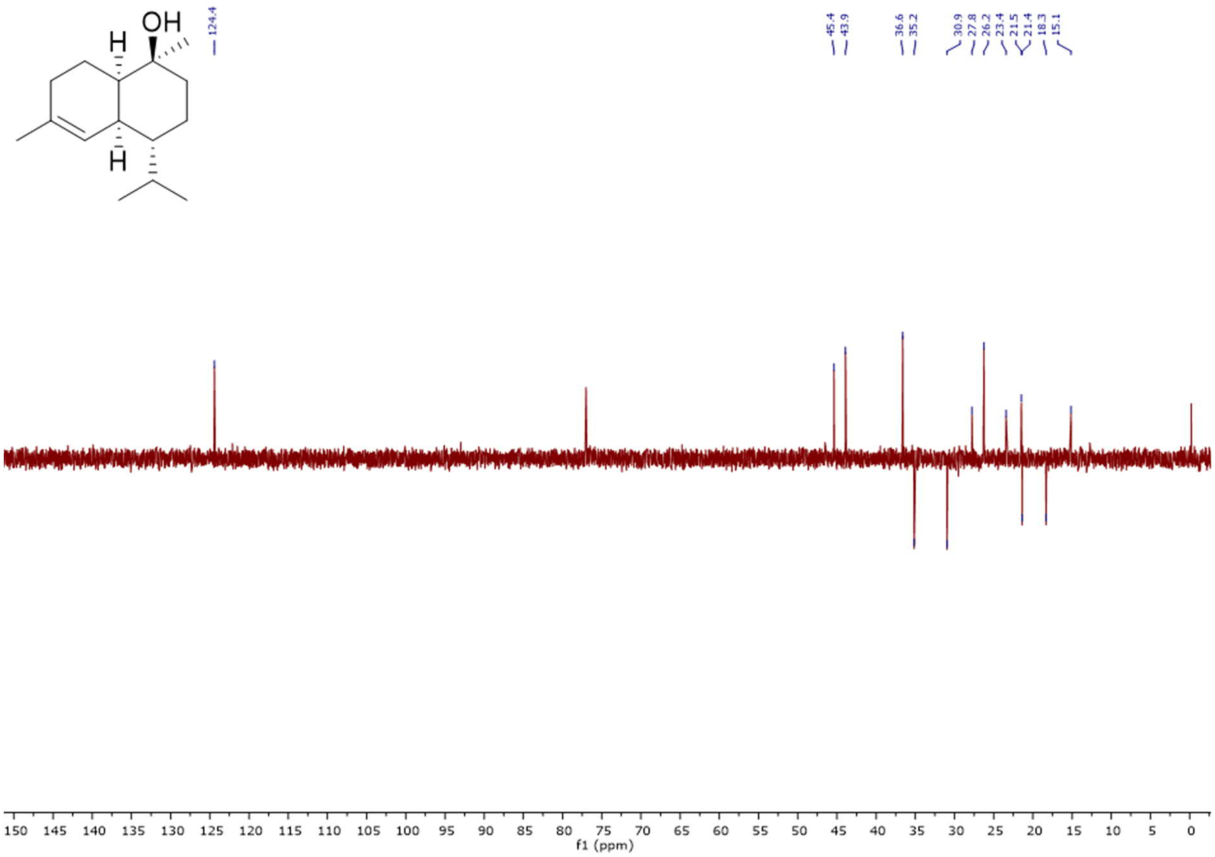

Figure S15: DEPT spectrum of δ-cadinol (**12**).

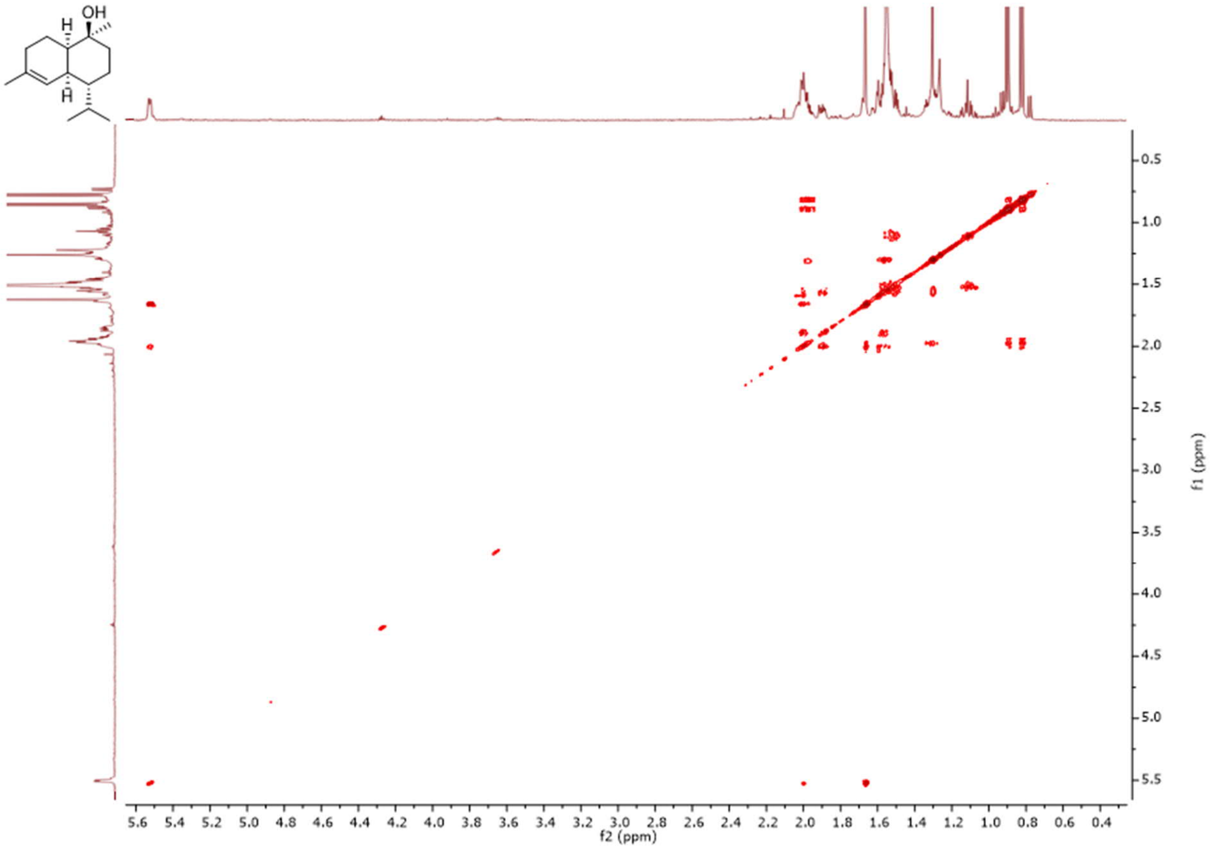

Figure S16: COSY spectrum of δ-cadinol (**12**).

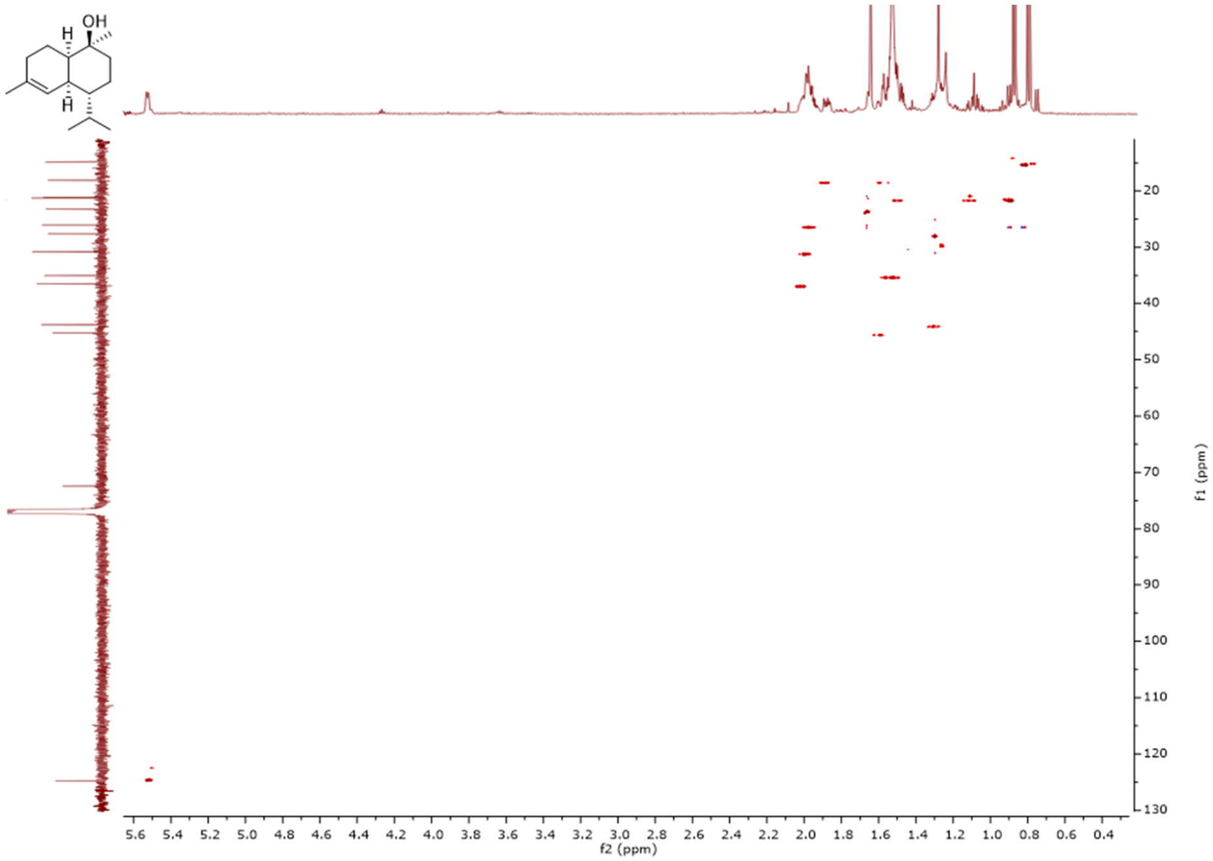

Figure S17: HSQC spectrum of δ-cadinol (**12**).

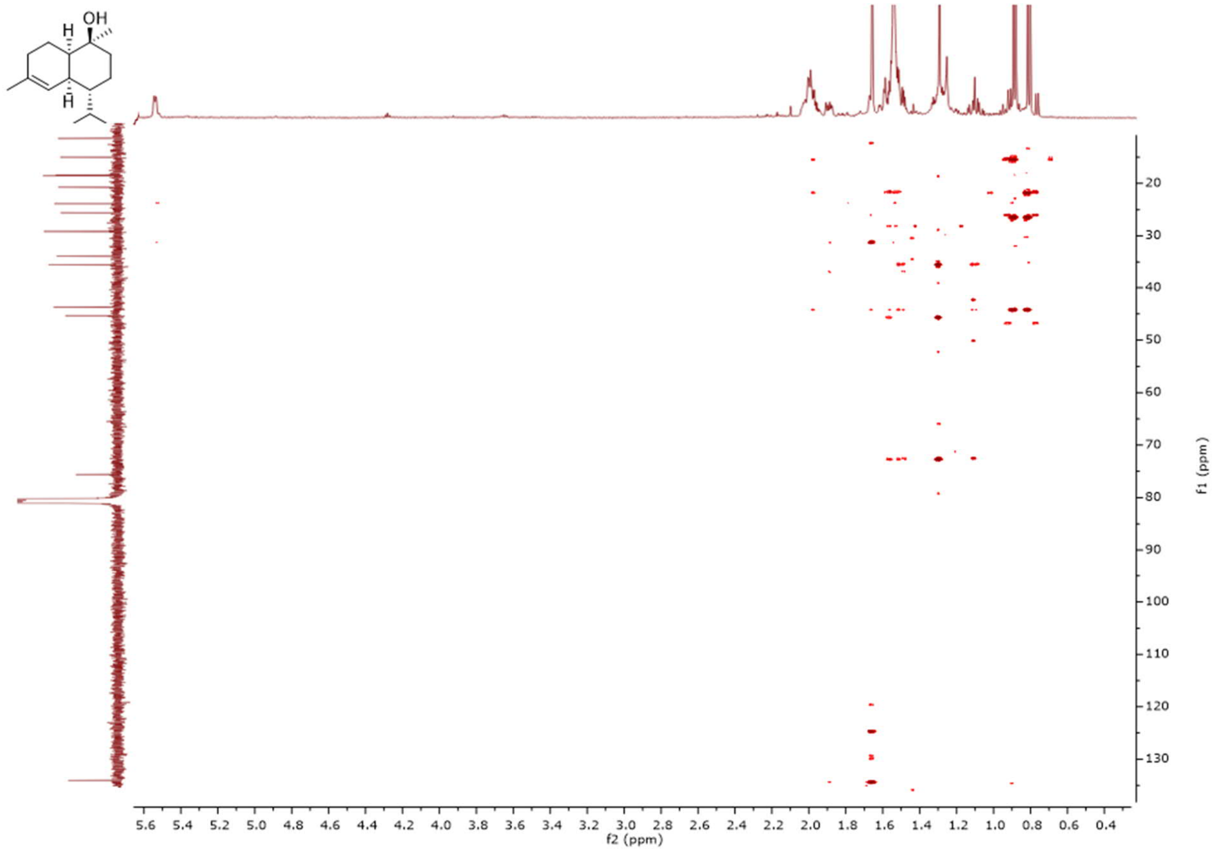

Figure S18: HMBC spectrum of δ-cadinol (**12**).

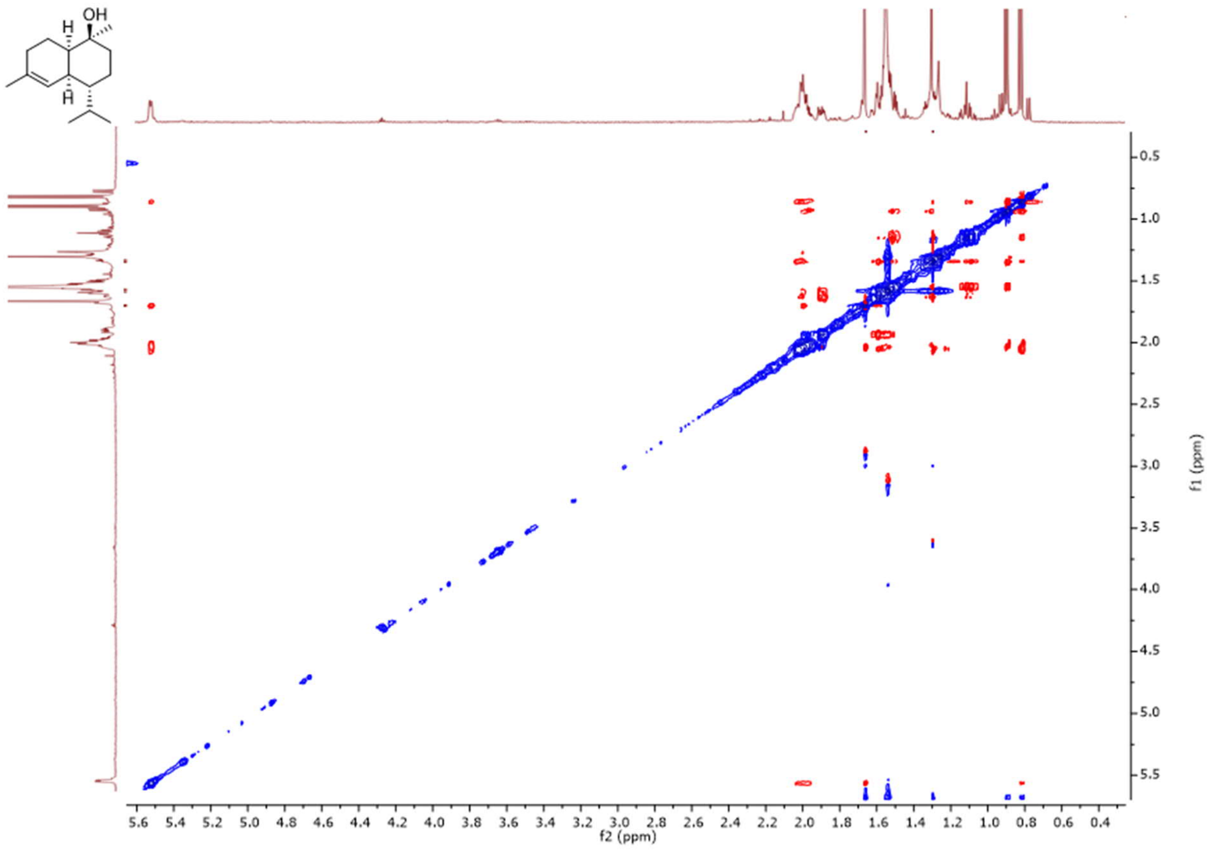

Figure S19: NOESY spectrum of δ-cadinol (**12**).
